# General Movements Assessment and Amiel-Tison Neurologic Examination in Neonates and Infants: Correlations and Prognostic Values Regarding Neuromotor Outcomes

**DOI:** 10.3390/life16010081

**Published:** 2026-01-05

**Authors:** Adrian Ioan Toma, Vlad Dima, Lidia Rusu, Andreea Necula, Roxana Pavalache Stoiciu, Larisa Andrășoaie, Andrada Mirea, Anca Roxana Bivoleanu

**Affiliations:** 1 Life Memorial Hospital, 010719 Bucharest, Romania; adrian.toma@prof.utm.ro (A.I.T.);; 2 Faculty of Medicine, University Titu Maiorescu, 040441 Bucharest, Romania; 3 Filantropia Clinical Hospital, Neonatology Department, 011132 Bucharest, Romania; 4 Faculty of Medicine, Department of Balneophysiokinesiotherapy and Rehabilitation, Carol Davila University of Medicine and Pharmacy, 050474 Bucharest, Romania; 5 Regional Centre of Public Health, 700465 Iasi, Romania; 6 National Clinical Centre for Neuropsychomotor Rehabilitation of Children “Robănescu-Pădure”, 041408 Bucharest, Romania; 7 Neonatal Intensive Care Unit, Clinical Hospital of Obstetrics and Gynaecology Cuza Vodă, 7000038 Iași, Romania

**Keywords:** general movements assessment, Amiel-Tison neurologic exam, cerebral palsy, delayed sitting, delayed walking, predictive value

## Abstract

**Background**: Our study aimed to investigate whether the general movements assessment (GMA) and Amiel-Tison Neurologic Examination performed at term-equivalent age (TEA) and 12 weeks corrected age (CA) could predict the occurrence of cerebral palsy (CP) and delayed gross motor milestones in a sample of term and preterm infants and whether the predictive values could be increased by using the two examinations during the same visit. **Methods**: A total of 70 infants (62 preterm and 8 at term) were examined at TEA and 12 weeks CA using GMs (General Movements) and Amiel-Tison (AT) examinations. We determined the correlation between the results of the examinations and several selected items (scarf sign, popliteal angle, and axial tone) and neuromotor outcomes (presence of cerebral palsy (CP), independent sitting, and independent walking). We attempted to build binary logistic regression models using items from both examination techniques to assess whether the combined use of the two exams could have a better predictive value than using one technique alone. **Results**: We analyzed the entire group and, separately, the subgroup of preterm infants. For the whole group, there was a statistically significant correlation between the GM examinations at TEA and 12 weeks CA (*p* < 0.008) as well as between the results of GM and AT exams performed at TEA (*p* < 0.001) and 12 weeks CA (*p* < 0.001). The strongest individual predictor for CP in the whole group was the absence of fidgety movements at 12 weeks CA (AUC (Area Under the Curve) = 0.873; CI (confidence interval) 95%: 0.748–0.998; *p* < 0.001) and a non-optimal result at the synthesis of findings of AT exam at 12 weeks CA (AUC = 0.755; CI 95%: 0.617–0.892; *p* < 0.003). In the subgroup of 62 premature neonates, absent fidgety movements at 12 weeks CA (AUC = 0.925; CI 95%: 0.819–1.031; *p* < 0.001) and a non-optimal result in the synthesis of findings of AT exam at 12 weeks CA (AUC = 0.772; CI 95%: 0.620–0.924; *p* < 0.005) were statistically significant predictors for the risk of CP. In the case of delayed sitting and delayed/absent walking, absent fidgety movements and non-optimal results in the AT exam at TEA and 12 weeks CA were the strongest individual predictors in the whole group as well as in the subgroup of preterm infants. The following exploratory logistic regression models reached statistical significance: one model combining absent fidgety + abnormal scarf sign + abnormal popliteal angle at 12 weeks CA for CP in the whole group (*p* < 0.032) and preterm infants (*p* < 0.034) and for delayed sitting in preterm infants (*p* < 0.041) and a second model combining absent fidgety + abnormal scarf + abnormal popliteal + abnormal axial + abnormal synthesis for CP risk (*p* < 0.046) and delayed sitting (*p* < 0.001) in preterm infants at 12 weeks CA. **Conclusions**: The absence of fidgety movements at 12 weeks CA represented the strongest individual predictor for the occurrence of CP and delayed gross motor milestones in our sample, both in the whole group and the subgroup of preterm infants. The combination of GM and AT findings reached statistical significance for the detection of CP risk in the whole group and CP risk and delayed sitting in the subgroup of preterm infants. Due to sample size limitations, the results should be regarded with caution, and larger studies are needed before robust conclusions can be drawn.

## 1. Introduction

Timely identification and referral for early intervention are an essential part of the management of NICU (Neonatal Intensive Care Unit) graduates at risk of cerebral palsy or motor impairment [1]. Even though an accurate diagnosis of cerebral palsy can usually be established around 18–24 months corrected age [2,3], studies have shown that early identification of infants at risk is possible using special clinical examination techniques [4].

Neurologic examinations of neonates and young infants can be performed using different tools and techniques [5], based on different concepts that will be briefly reviewed below.

Firstly, neurologic exams can be performed classically, as a formal neurologic examination similar to the neurologic examination of older children and adults but considering the maturational features of neonates and young infants, as suggested by Volpe [6]. They are based on the classic concepts of Sherrington regarding the reflex arc, excitation, and response—a normal response will imply the integrity of the pathway, while an abnormal or absent response will suggest an abnormality [7].

There are other examination techniques using the same theoretical basis of response to an external stimulus but specifically developed for neonates and infants using tone as the main feature: the Hammersmith Neonatal Neurologic Examination [8] and the updated Amiel-Tison Neurological Assessment at Term (ATNAT) [9,10]. The interpretation of these examinations is based on the optimality concept [11,12], i.e., it is easier to predict a normal outcome following a normal examination. To ensure consistent results, in the case of preterm neonates, examinations are performed at 40 weeks corrected age (Term Equivalent Age—TEA) [8,9,10]. The physiological basis of the Amiel-Tison neurologic examination is the concept of two control systems for motor function. The subcortical system, based in the cerebellum and brainstem, matures in a caudal-to-cranial manner and controls the flexor tone and anti-gravity maintenance of posture. The cortical (pyramidal) system, based in the hemispheres and basal ganglia, matures in a descending cranial-to-caudal pattern and is responsible for the progressive acquisition of motor control, such as head control, sitting, and walking, during the first year of life [9,10,12]. At 40 weeks corrected age (term-equivalent age), the superior system begins to take power (*prise de pouvoir*—in French) from the inferior subcortical system, at the level of the flexor and extensors of the neck. This allows the active tone—*motricité dirigéé*—to be tested by placing the body in a certain position and expecting a certain response in the tone. The pull to sit and reverse manoeuvres elicit contractions of the flexors and extensors of the neck, obtaining a balance that results in normal situations in the maintenance of the head control for a few seconds [12,13]. The exam includes items structured in several domains: cranial exam, neurosensory (ocular and hearing), spontaneous motor activity, passive tone (upper limbs, lower limbs and trunk)—the angle between two segments (popliteal angle), the displacement with a controlled force from a certain point (scarf sign) or recoil (arms or legs), active tone (see above), archaic reflexes, palate and tongue, toleration to manipulation, and feeding autonomy. There are also noted special circumstances for the exam and the medical status of the patient [10,12]. Each item receives a certain score; there is no final score, though. Based on the predominance of normal or abnormal signs, the exam is scored as normal or abnormal (moderately or severely abnormal) [10,12]. This examination technique has the advantage of having versions that can be used in infants and toddlers and has the same principles [13]. The performance of the exam does not require formal training, although it has specific administration guidelines [5,9,10] and has been noted to have a good inter-observer reliability for most of the items [14]. Regarding the predictive value for the incidence of CP and motor dysfunction, a study performed on term neonates showed that all infants with optimal ATNA (Amiel-Tison Neurological Assessment) scores had normal neurological examinations on follow-up. None of the neonates with a severely abnormal examination had a normal neurological exam at follow-up; thus, the agreement between ATNA and the exam at follow-up was excellent (K = 0.83) [15]. In the case of very preterm infants, the combination both results from ATNAT and head ultrasound had the best predictive value. When using the neurologic exam alone, the probability of a normal outcome at 12 months was 94% [16].

The general movements assessment (GMA) proposed by Precthl uses a different paradigm [17]. This technique consists in the evaluation of spontaneous movements in the neonates and small infants, by the use of observation and videotaping [17,18]. The physiological basis of the method is that the normal general movements are spontaneous movements of the whole body, generated by a group of subcortical neurons (central pattern generators, CPGs) [19] and modulated by the cortical structures [20,21]. The lack of modulation by the cortical structures due to different lesions and pathologies results in an abnormal GM pattern [17,18,19,20,21]. The movements involve the entire body, with a variable sequence of movements of the head, neck, trunk, arms, and legs. Those movements are defined by a gradual beginning and end and variable amplitude, intensity, and speed [17]. Certain types of GMs are observed at different postnatal ages. In the neonatal period, until the age of 6–9 weeks, the normal movement pattern, called writhing movements, consists of movements involving the whole body, waxing and waning in intensity, force and speed, with fluent and elegant rotations of the limbs and trunk that create an impression of variability [17]. The abnormal movements in the neonatal period are either poor repertoire movements (monotonous movements that do not occur in a complex way) or cramped–synchronized movements (the limbs and trunk muscles contract and relax almost simultaneously) [17]. At 6–9 weeks, the normal pattern of movements changes to fidgety movements (movements of small amplitude, moderate speed, and variable in acceleration, probably representing fine-tuning of the proprioceptive system that occurs in the limbs, trunk and neck) [17]. The absence of fidgety movements is considered an abnormal pattern [17]. The GM disappear around 20 weeks [17,18] post-term when the voluntary movements appear; thus, the GMA examination could be used until approximately 20 weeks corrected age. Performing and interpreting the exam requires formal training under the tutorship of GM Trust. Since the introduction of the examination technique, it has been observed that GM is a good predictor for the occurrence of CP [17], and is considered the most valuable test in the first 3 months of life [2]. In a cohort of preterm neonates, it has been shown that the observation of the GM pattern was 100% sensitive for the identification of the risk of CP, and a cramped–synchronized GM pattern had a sensitivity of 92.5% and a specificity of 100% when it came to identifying the risk of CP [22]. A study performed on 130 patients showed that a persistent CS pattern and the absence of fidgety movements were strong predictors for the appearance of CP [23]. Another later study on a large cohort of infants from different continents showed that 95% of the children who later developed CP did not show fidgety movements [24].

Two studies compared the concordance of the two methods (GMA and Amiel-Tison Examination) regarding the prediction of the neurologic and developmental outcomes. A study performed on 45 preterm infants, followed for 12–15 months corrected age, showed the agreement between the two methods was excellent in the case of the examinations performed at term (k = 0.87) and good at in the case of the exams performed at 3 months (k = 0.54); the sensitivity was 92–100% for the Amiel-Tison exam and 96–100% for the GMA at term and 3 months [25]. In another study involving 103 very-low-birthweight infants regarding the outcome at 20 months, the GM exam performed at TEA had a positive predictive value of 89% and a negative predictive value of 84% and the Amiel-Tison exam had a positive predictive value of 33% and a negative predictive value of 88% for the identification of the outcomes [26].

The idea to combine the findings of two neurologic examinations in the same patients in order to increase the accuracy and the predictive value was found to be successful. An Italian group combined an exam based on classical neurologic examination principles focused on tone (HINE—The Hammersmith Infant Neurological Examination) with GMA, and the result has been an improvement in the early prediction of the neurodevelopmental outcome [27]. To date, no studies have assessed the combined findings of the ATNAT [9,12] or Amiel-Tison Neurologic Examination of the Infant and Children [13] and GMA [17,18] in the case of the follow-up of the NICU graduates. Thus, the research objectives of our group were as follows:

- To identify the correlations between the normal and abnormal results of the GMA and Amiel-Tison neurologic examinations performed in a population of NICU graduates at 40 weeks corrected age (Term Equivalent Age—TEA) and 12 weeks corrected age.
- To identify the prognostic value of the two examinations and the different items used for detecting the risk of cerebral palsy or the risk of gross motor delay (independent sitting and independent walking).
- To verify by, using logistic regression models, if the combination of results from the two examinations at term-equivalent age and 12 weeks corrected age results in a better prognostic value than using the result of each examination separately.

We aimed to test these hypotheses in a sample of term and preterm NICU graduates and in a subgroup of former preterm neonates from the whole population sample.

## 2. Materials and Methods

### 2.1. Population Studied

The study was performed within the follow-up program of Medlife Medical Park, Bucharest. Approval was obtained according to the regulations from the Ethics Committee of MedLife Medical System (Decision No 1097/15 March 2023 renewed with decision No 11/19 November 2025). Informed consent was obtained from the parents of the patients.

**Figure 1:**
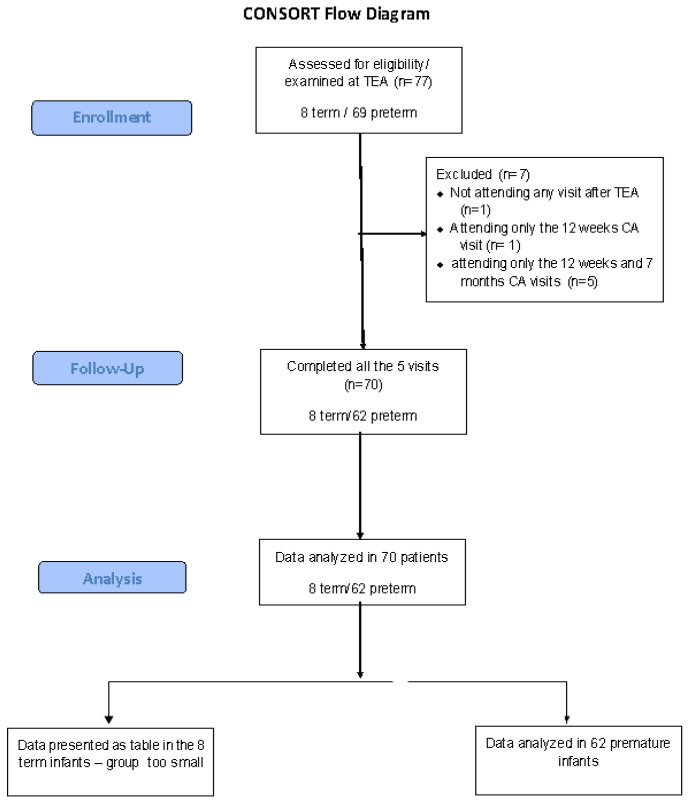
Consort flow diagram of the sample studied.

The sample of patients studied was represented by 70 neonates—62 preterm and 8 term neonates with different conditions (see the Results Section), who were consecutively admitted to our program at their visit at TEA and fulfilled the criteria of being examined at TEA, 12 weeks, 7, 12, 18 and 24 months CA. In the case of the term neonates, all the patients fulfilled the 5 mandatory visits. In the case of preterm infants, 62/69 fulfilled the 5 visits and were considered for the final analysis. Seven patients were lost to follow-up—six fulfilled the TEA and 3-month visit, and five out of seven also came back for the 7-month visit. At the visit at 12 months corrected age, 62/69 came for the visit—after 12 months, no other patient has been lost to follow-up (consort chart, Figure 1). The patients were born in different units in Bucharest and in the country and were referred to the follow-up program of our clinic by their attending physicians. We attempted to enroll all 77 infants that presented to our program within the study period, but we are not aware of the number of infants fulfilling the inclusion criteria in all the units that sent patients during that time interval, or of the number of infants sent by the attending physicians to be enrolled in our program during the same time period. The initial non-enrolment could represent an additional source of selection bias. The inclusion criteria were as follows:

- The patient belongs to a category of neonates at risk that needed to be included in the follow-up program for high-risk neonates, according to the guidelines of Romanian Neonatology Association [28], consistent with the international guidelines in the field [29].
- The informed consent of the family has been obtained.
- The patient was followed until 24 months corrected age or until a diagnosis of cerebral palsy (CP) was established—whichever occurred sooner.

The recruitment for the study started in June 2023 and ended in October 2023. The patients were followed until October 2025, when the last patient attained the 2-year CA milestone.

### 2.2. Neurologic Examinations

The protocol of the neurologic examination can be followed in Table 1.

At TEA and 12 weeks corrected age, we performed GMA assessment [17,18] and Amiel-Tison examinations corresponding to the age of the infant (ATNAT at TEA [9] or Amiel-Tison Neurologic Examination for the infant and child aged 0–6 years at 12 weeks CA) [13]. In the case of the subsequent visits (7,12,18, and 24 months CA), an Amiel-Tison examination with the appropriate items for the age was performed [13]. To avoid an interpretation bias, the GM and Amiel-Tison examinations were performed by different persons. The person performing the GM examinations (ARB) received appropriate training from the GM Trust, while the person performing AT examinations received training in Port Royal Hospital Paris and had 20 years of experience in this examination (AIT). Notably, he was also trained in GMA.

In the case of the General Movements Assessment (GMA) at TEA, the movement pattern was noted, according to the classic guidelines:

- Normal (writhing pattern): movements of small-to-moderate amplitude, slow-to-moderate speed, involving in a variable sequence the head, neck, trunk, arms, and legs, with an elliptical form [17,18];
- Poor repertoire pattern (PR): movements that are monotonous in character and do not occur in a complex way [17,18];
- Cramped–synchronized pattern (CS): all limbs and muscles contract and relax almost simultaneously; the movements lack the normal smooth and fluent character and are rigid [17,18].

In the case of the GMA exam performed at 12 weeks CA, the presence or absence of the fidgety movements was noted. Fidgety movements, the normal GMA pattern at that age, consist of movements of small amplitude, at moderate speed and variable acceleration of the neck, trunk, and limbs in all directions continually [17,18].

In the case of ATNAT and the Amiel-Tison neurologic examination of the infant and child, the exam has been scored as optimal and non-optimal based on the guidelines provided by the authors. An optimal result is represented by the absence of the neurologic signs, while a non-optimal result consists of the presence of neurologic signs (abnormalities of tone and excitability noted with 1 or 2 in the examination chart) [9,12,13]. The scores for certain individual items that were considered important were noted separately to be investigated for prognostic values (also graded as normal and abnormal according to the examination charts) [9,12,13]. Three items were selected as representative for the passive tone of the upper limbs (scarf sign), passive tone of the lower limbs (popliteal angle), and active tone (pull-to-sit and reverse maneuver) [12,13] (Table 2).

Regarding the actual procedure for scheduling the visits, all the appointments were organized according to the calendar for follow-up—see above (Table 1). The following visit was programmed at the end of each consult. The patients could reschedule the visit themselves, by phone call, within a two-week interval from the programmed visit. If they did not come to the programmed visit, we tried to reschedule by a phone call, and if they did not answer or did not come to the visit, they were considered lost to follow-up.

### 2.3. Outcome Measures

The outcome measures of the study were represented by the occurrence of independent sitting, independent walking and a diagnosis of cerebral palsy. In the case of the independent sitting, we noted both the presence of the gross motor acquisition and the age of occurrence/defined as normal according to the examination guidelines of Amiel-Tison and Gosselin [13].

Independent sitting has been defined as the capacity to sit without support for at least 15 s [13]. The optimal age of acquisition was considered to be before 9 months CA; if independent sitting occurs after that age, it is considered delayed [13].

Independent walking has been defined as the capacity to take a minimum of 5 unaided steps [30,31]. The optimal age of acquisition was considered before 18 months CA. If independent walking occurs after that age, it is considered delayed [13].

The ages of attainment of the motor milestones, including sitting and walking, were not based on parental recall, but on whether the milestone was present or absent at scheduled visits. Classification as “delayed sitting” (beyond 9 months CA) and “delayed walking” (beyond 18 months CA) was operationalized based on absence at the nearest scheduled visits (7/12 months for sitting, 12/18/24 months for walking).

Cerebral palsy is diagnosed according to the 2006 definition as a permanent disorder of movement and posture causing activity limitation that is attributed to non-progressive disturbances occurring in a developing brain [32]. The diagnosis is established by the neurologist as soon as possible, but usually not before 12 months of age. CP cases are classified according to the European CP initiative as spastic, dystonic, or ataxic, with unilateral or bilateral involvement, and with predominance in the upper or lower limbs, or all four limbs equally involved [33]. The GMFCS (Gross Motor Function Classification System) grade is also established as soon as possible, usually at the age of 2 years CA [2].

### 2.4. Statistical Analysis

In order to avoid interpretation bias [34], the statistical analysis was performed by a person from another center, with no medical background, who had not been part of the data collection team (LR). The statistician received the anonymized database together with the research questions.

The data were centralized in a SPSS 18.0 database and processed with the statistical functions for which they are suitable, at a significance threshold of 95%.

The variables investigated (independent and outcome variables) were of a binary type (usually having two possible values—normal and abnormal). In order to assess the association between the results of the examinations and the risk of CP, a delayed sitting and delayed/absent walking Chi-Square test was used [35].

The analysis of the prediction was performed for the entire study sample (70 patients) and for the subgroup of preterm neonates (62 patients).

In order to investigate the value of different signs and results of the examination in prediction of the outcome variables, we used the Odds Ratio and the ROC curve—a visual representation of model performance that allowed us to identify the strongest predictors. Collinearity diagnosis was performed for all the variables and found values of VIF (variance inflation factor) <5 for CP, sitting, and walking, showing that the multicollinearity does not influence the variance of the regression coefficients.

In order to predict the probability of the binary outcomes, we attempted to use binary logistic regression models [36]. The stepwise forward selection method was used to build the models. For each statistics model, there was calculated the Odds Ratio (Exp (B)) for each predictor. We considered a model for which the *p*-value was <0.05 to be statistically significant.

## 3. Results

### 3.1. General Characteristics of the Population

As noted above, the study included only the infants who fulfilled all the visits until 2 years of age. Thus, the study cohort comprised 70 infants, of whom 62 were preterm and 8 had other conditions not related to prematurity and were almost all born at term (see below). The gender ratio was 30 male/40 female. Forty-five of the infants were singletons, twenty two were twins (eleven pairs), and there was one set of triplets (3 female neonates). The mean gestational age was 33.9 weeks, with a median of 34 weeks (standard deviation ± 2.835 weeks), minimum gestational age of 27 weeks and a maximum of 40 weeks. The overall incidence of CP was 15/70 in the whole group, with an incidence of 11/62 in the preterm neonates group and 4/8 in the group of neonates born at term.

**Figure 2:**
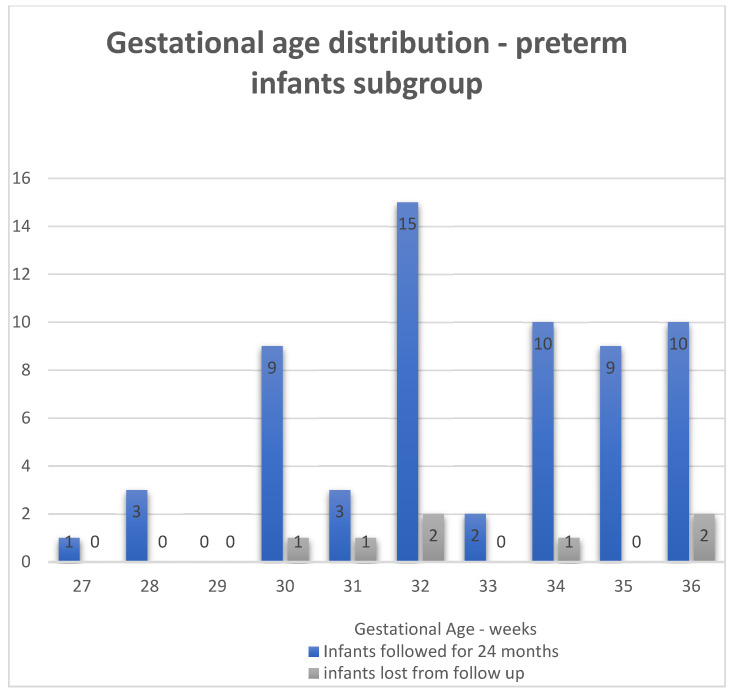
Distribution by gestational ages—subgroup of preterm infants.

The distribution by gestational ages of the subgroup of preterm infants can be seen in Figure 2. They are plotted as separate groups: the infants that were followed until 24 months corrected age and the infants lost to follow-up.

There has been a 100% rate of return for the neonates at term and 89.85% (62/69) in the preterm group. In the case of preterm infants lost to follow-up, the baseline characteristics did not differ significantly from the infants that completed the follow-up until 2 years: a mean gestational age of 33 weeks with standard deviation ± 2.94 weeks for the infants lost to follow-up, 32.82 weeks preterm infants that completed the follow-up, gender ratio: three male/four female in the group of patients lost to follow-up (not significantly different from the preterm infants group (24 male/38 female). During the follow-up process, at 12 weeks CA, 5/6 presented with fidgety movements and optimal results for the AT examination and at 7 months, 4/5 exhibited independent sitting and normal AT exam and 1/5 presented with a popliteal angle < 90°—this was reported for the same patient that did not present with fidgety movements at 12 weeks CA.

The non-preterm group was composed of infants with malformations and genetic conditions; the characteristics of the group could be examined in Supplementary Material Table S1. Due to the small size of the group, a statistical analysis was not performed.

### 3.2. GMA Examination at TEA and 12 Weeks Corrected Age—Analysis of the Whole Group

At the GMA examination performed at TEA, 16 patients presented with a normal GM pattern, 37 with a poor repertoire (PR) pattern, and 17 with a cramped–synchronized (CS) pattern. At the examination performed at 12 weeks CA, in 55/70 patients, the fidgety movements were present, and in 15/70, fidgety movements were absent.

Even if the analysis of the whole group is presented in the following section, as stated above, it should be acknowledged that the whole group lacks homogeneity, and even if the number of non-preterm infants (patients with congenital and genetic conditions) is small, the heterogeneity of the group should result in caution when analyzing and generalizing the results. The decision to analyze the whole group first has been motivated by the fact that we attempted to investigate the correlation of the examinations and the predictive value in a sample consisting not only of preterm infants but also of infants of different gestational ages and pathologies in order to increase the value of the results. Also, this was our initial study protocol, and not analyzing the whole sample could be considered to cause selection bias. Accordingly, we decided to analyze the whole group and to acknowledge the limitations in this case.

There has been a very good correlation between the GM patterns at TEA and 12 weeks CA (Table 3), with all the patients with normal GM patterns at TEA progressing to a pattern with present fidgety movements and 6/17 of the patients with CS and 9/37 patients with PR showing no fidgety movements at 12 weeks CA.

When separately analyzed, the GM patterns at 40 weeks GA did not have a statistically significant association with the occurrence of CP. The absence of the fidgety movements at 12 weeks CA was a statistically significant predictor for the appearance of CP (Table 4).

Regarding delayed sitting (Table 5) and delayed/absent walking (Table 6), a statistically significant association has also been found in the case of absent fidgety movements.

### 3.3. Amiel-Tison Neurologic Evaluation at TEA and 12 Weeks CA—Whole Group

The ATNAT performed at TEA was considered optimal in 47/70 cases. The result was considered suboptimal in 23/70 cases. The Amiel-Tison neurologic examination at 12 weeks CA had an optimal result in 42/70 cases and was graded as non-optimal in 28/70 infants.

Both the final result (synthesis) of the ATNAT and the values of the items (scarf sign, popliteal angle, axial tone) had a statistically significant association with the occurrence of cerebral palsy and delayed/absent walking (Table 4 and Table 6). In the case of the delayed sitting, an abnormal popliteal angle at TEA has been associated with delayed sitting (Table 5), while the other items of ATNAT did not show any statistically significant association with this delay.

In the case of the Amiel-Tison evaluation performed at 12 weeks CA, we have also identified statistically significant associations between the final result/synthesis and the selected items and the appearance of CP and delayed walking (Table 4 and Table 6). As in the case of ATNAT, an association with delayed sitting has been observed in the case of the the final result (synthesis) of the exam findings (Table 5), in addition, a statistically significant association has been found in the case of abnormal axial tone.

### 3.4. Correlation Between GMA and Amiel-Tison Examinations—Whole Group

In the case of the examinations performed at TEA, there is a statistically significant correlation between the GM patterns and an abnormal popliteal angle, abnormal axial tone and the result (synthesis) of the AT examination (synthesis) (Table 7).

In the case of the examinations performed at 12 weeks CA in the whole group of patients, there has been a good correlation between the GM and Amiel-Tison examinations, both in the case of the synthesis of the results and of the selected items (Table 8).

### 3.5. Logistic Regression Models

#### 3.5.1. Logistic Regression Models—Whole Group

**Figure 3:**
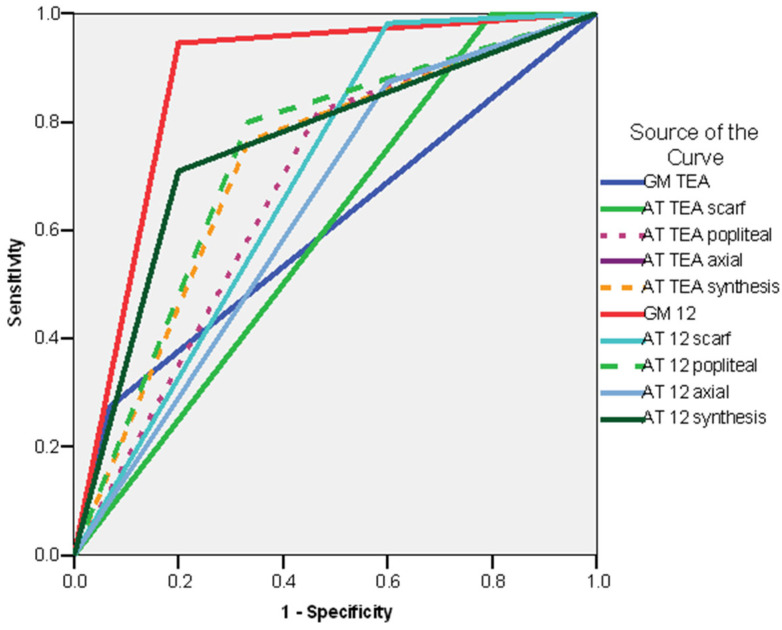
ROC curve. Risk of CP. Whole group legend: AT TEA—Amiel-Tison exam at Term Equivalent Age; AT 12—Amiel-Tison exam at 12 weeks corrected age; GM TEA—general movements assessment at Term Equivalent Age. GM 12 general movements assessment at 12 weeks corrected age.

In order to investigate which items from the examinations could be statistically significant predictors for the risk of cerebral palsy (CP), we calculated the odds ratios (Supplementary Material Table S2.1) and the ROC curves (Figure 3) for the different GM patterns in the case of GMA, for the selected items and final results (synthesis) in the case of the Amiel-Tison examinations. In the case of the GM patterns at TEA, we compared the patients with a normal pattern with those with an abnormal one (cramped–synchronized and poor repertoire). In the case of the GM examination at 12 weeks CA, we compared the patients with fidgety movements with those in whom the fidgety movements were not present. In the case of Amiel-Tison’s examination at TEA and 12 weeks CA, we compared the patients with normal/optimal findings with the patients with abnormal/non-optimal results.

The ROC curve for the risk of CP of the whole group showed that all the variables had an AUC > 0.6000, with the strongest individual predictor being the GM pattern at 12 weeks CA (AUC = 0.873; CI 95%: 0.748–0.998; *p* < 0.001), followed by the synthesis of the Amiel-Tison exam at 12 weeks CA (AUC = 0.755; CI 95%: 0.617–0.892; *p* < 0.003), Amiel-Tison exam at 12 weeks CA—popliteal angle (AUC = 0.733; CI 95%: 0.580–0.887; *p* < 0.006), and the synthesis of the Amiel-Tison exam at TEA (AUC = 0.715; CI 95%: 0.561–0.869; *p* < 0.011) (Figure 3).

In order to check if a combination of items from the two examinations resulted in a better prediction for the risk of CP than each examination alone, we attempted to develop binary logistic regression models for the exams at TEA and 12 weeks CA. The models can be found in Appendix A, Table A1—Exams at TEA; and Table A2—Exams at 12 weeks CA. As previously stated, given the sample size and event counts, these multivariable models are exploratory and prone to overfitting. In the case of risk of CP, the predictive models showed statistical significance only for the independent variables GM pattern at 12 weeks (absent fidgety movements) + AT12 weeks scarf sign (abnormal) and AT12 weeks popliteal angle (abnormal) (*p* < 0.032) (model 3 Table A2—Appendix A).

Similar models of risk were attempted for the delayed acquisitions of gross motor skills (sitting and walking).

In the case of delayed sitting, significant odds ratios were found for the abnormal popliteal angle at TEA and for absent fidgety movements and abnormal axial tone and non-optimal Amiel-Tison examination (synthesis) at 12 weeks CA (Supplementary Material—Table S2.2).

**Figure 4:**
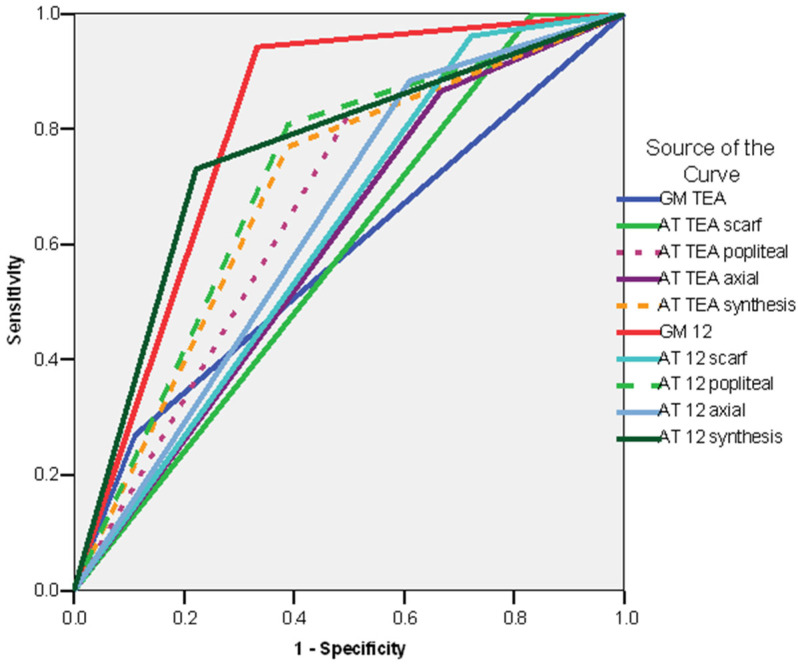
ROC curve. Risk of delayed sitting—whole group legend: AT TEA—Amiel-Tison exam at Term Equivalent Age; AT 12—Amiel-Tison exam at 12 weeks corrected age; GM TEA—general movements assessment at Term Equivalent Age. GM 12 general movements assessment at 12 weeks Corrected Age.

The ROC curve showed the following good predictors of delayed sitting (AUC > 0.600): GM examinations at 12 weeks CA—absent fidgety movements (AUC = 0.804; CI 95%: 0.666–0.943; *p* < 0.001), synthesis of AT exam at 12 weeks CA (AUC = 0.754; CI 95%: 0.622–0.886; *p* < 0.001), AT exam at 12 weeks CA—popliteal angle (AUC = 0.709; CI 95%: 0.561–0.858; *p* < 0.008), synthesis of the AT exam at TEA (AUC = 0.690; CI 95%: 0.541–0.839; *p* < 0.017), AT exam at TEA—popliteal angle (AUC = 0.663; CI 95%: 0.508–0.819; *p* < 0.040) (Figure 4).

The binary logistic regression models built for the risk of delayed sitting, both at TEA (Table A3) and 12 weeks CA (Table A4), did not show any predictive value.

In the case of delayed/absent walking, the odds ratios were significant for GM evaluation at 12 weeks CA, abnormal scarf sign at TEA and 12 weeks CA, abnormal popliteal angle at TEA, and 12 weeks CA and abnormal axial tone and synthesis of the Amiel-Tison exam, both at 12 weeks CA (Supplementary Material S2, Table S2.3).

**Figure 5:**
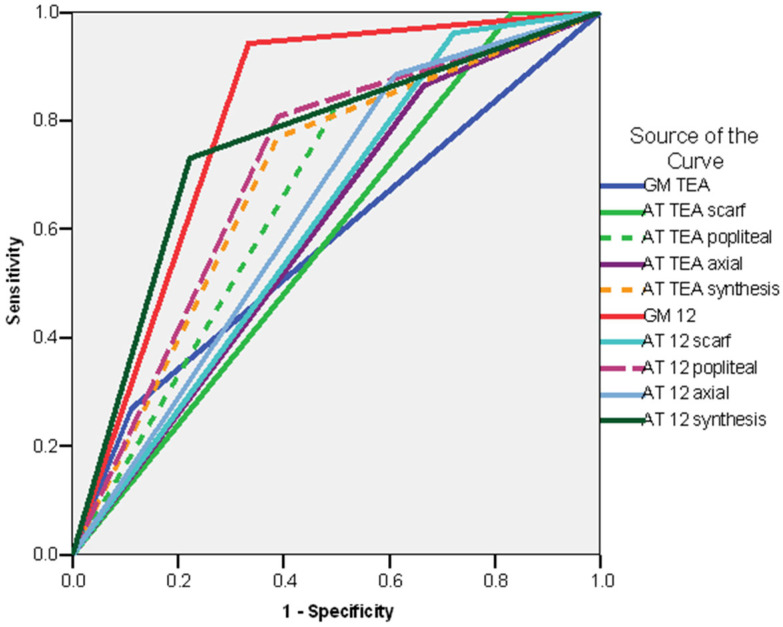
ROC curve—delayed/absent walking—whole group. Legend: AT TEA—Amiel-Tison exam at Term Equivalent Age; AT 12—Amiel-Tison exam at 12 weeks corrected age; GM TEA—general movements assessment at Term Equivalent Age. GM 12 General movements assessment at 12 weeks corrected age.

The ROC curve revealed the following factors as good predictors of delayed/absent walking (AUC > 0.600): GM 12 weeks CA—absent fidgety movements (AUC = 0.804; CI 95%: 0.666–0.943; *p* < 0.001), synthesis of the AT exam at 12 weeks (AUC = 0.754; CI 95%: 0.622–0.886; *p* < 0.001) and AT exam at 12 weeks CA—popliteal angle (AUC = 0.709; CI 95%: 0.561–0.858; *p* < 0.008), synthesis of the AT exam at TEA (AUC = 0.690; CI 95%: 0.541–0.839; *p* < 0.017), AT exam at TEA—popliteal angle (AUC = 0.663; CI 95%: 0.508–0.819; *p* < 0.040) (Figure 5).

We did not identify any statistically significant binary logistic regression models in the case of delayed/absent walking for the whole group (Table A5—examinations at TEA and Table A6—examinations at 12 weeks CA).

#### 3.5.2. Logistic Regression Models—Subgroup of Preterm Infants

Subsequently, we attempted to build logistic regression models for the risk of CP, delayed sitting, and delayed/absent walking in the subgroup of the 62 preterm infants.

In the case of risk of CP, the odds ratios can be viewed in Table 9. The following statistically significant predictors were found for the GMA—absent fidgety movements at 12 weeks CA (*p* < 0.001), for the AT exam at TEA—abnormal scarf sign, abnormal popliteal angle, non-optimal result at the synthesis of the exam (*p* < 0.002). In the case of the AT exam at 12 weeks CA, an abnormal scarf sign and popliteal angle and the abnormal synthesis were found to be statistically significant predictors (*p* < 0.001).

**Figure 6:**
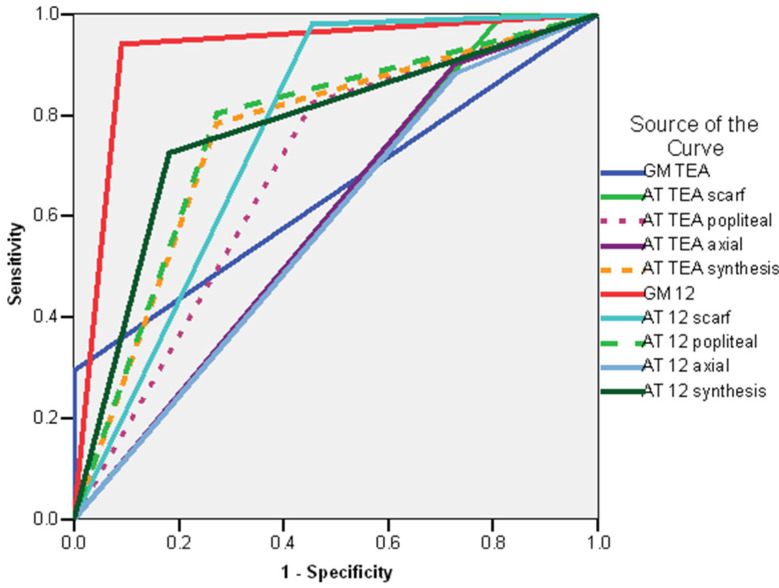
ROC curve. Risk o—Am f CP in the preterm infant subgroup. Legend: AT TEAiel-Tison exam at Term Equivalent Age; AT 12—Amiel-Tison exam at 12 weeks corrected age.; GM TEA—general movements assessment at Term Equivalent Age. GM 12 general movements assessment at 12 weeks corrected age.

In the subgroup of preterm neonates, the ROC curve revealed the following variables to be good predictors for CP (AUC > 0.600): the absence of fidgety movements at the GM exam at 12 weeks CA (AUC = 0.925; CI 95%: 0.819–1.031; *p* < 0.001), the synthesis of the AT exam at 12 weeks CA (non-optimal/abnormal) (AUC = 0.772; CI 95%: 0.620–0.924; *p* < 0.005), AT exam at 12 weeks CA—popliteal angle (abnormal) (AUC = 0.766; CI 95%: 0.599–0.932; *p* < 0.006), AT exam at 12 weeks CA—scarf sign (abnormal) (AUC = 0.763; CI 95%: 0.572–0.954; *p* < 0.007), the synthesis of the AT exam at TEA(non-optimal/abnormal) (AUC = 0.756; CI 95%: 0.589–0.923; *p* = 0.008), and AT exam at TEA—popliteal angle (abnormal) (AUC = 0.684; CI 95%: 0.495–0.874; *p* < 0.05) (Figure 6).

The tables of binary regression models for the risk of CP for the subgroup of preterm infants can be consulted in Appendix A (Table A7—examinations at TEA and Table A8—examinations at 12 weeks CA). No statistically significant model has been identified in the case of the examinations at TEA; two models that reached statistical significance have been identified at 12 weeks CA—one of the models included all the parameters investigated: GM exam at 12 weeks CA (absent fidgety), AT exam at 12 weeks CA—scarf sign (abnormal), AT exam at weeks CA 12—popliteal angle (abnormal), AT exam at 12 weeks CA—axial tone (abnormal) and the synthesis of the AT exam at 12 weeks CA (non-optimal) (*p* = 0.046; model 5). The odds ratio (Exp(B)) = 16.699, CI 95% = 1.851–327.783.

In the case of delayed sitting, the odds ratios can be observed in Table 10. The only two items associated with a statistically significant risk were the GM pattern at 12 weeks CA (*p* < 0.007) and AT exam synthesis at 12 weeks CA (*p* < 0.048).

**Figure 7:**
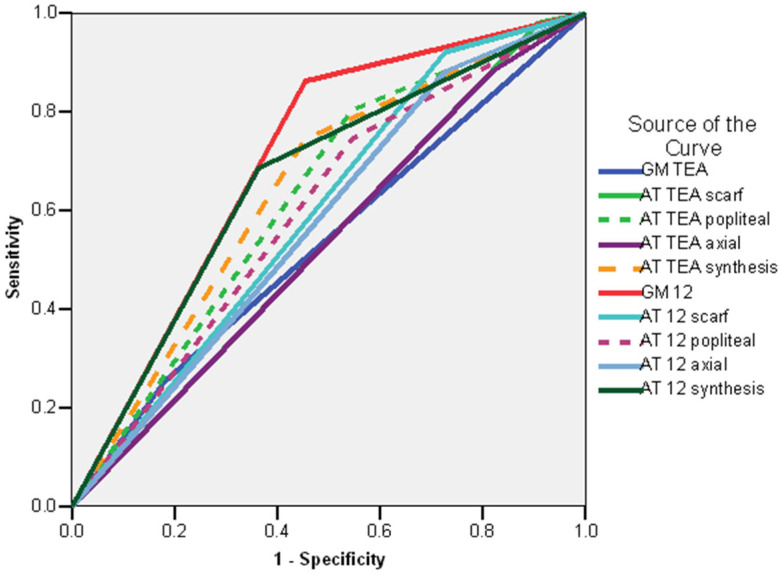
ROC curve. Risk of delayed sitting—subgroup of preterm infants. Legend: AT TEA—Amiel-Tison exam at Term Equivalent Age; AT 12—Amiel Tison exam at 12 weeks corrected age; GM TEA—general movements assessment at Term Equivalent Age. GM 12 general movements assessment at 12 weeks corrected age.

The ROC curve (Figure 7) confirms the following factors as good predictors for delayed sitting (AUC > 0.600): GM exam at 12 weeks CA—absence of fidgety movements (AUC = 0.704; CI 95%: 0.515–0.893; *p* < 0.035). It showed no significance for the synthesis of the AT exam at 12 weeks (AUC = 0.661; CI 95%: 0.480–0.843; *p* < 0.095), the AT exam at 12 weeks CA—popliteal angle (AUC = 0.600; CI 95%: 0.407–0.793; *p* < 0.302), the synthesis of the AT exam at TEA (AUC = 0.645; CI 95%: 0.457–0.834; *p* < 0.133), and the AT exam at TEA—popliteal angle (AUC = 0.629; CI 95%: 0.435–0.823; *p* < 0.182).

The tables of binary logistic regression models for the delayed sitting—subgroup of preterm infants, can be consulted in the Appendix A (Table A9—TEA and Table A10—12 weeks CA). No statistically significant model has been identified for the exam at TEA. At 12 weeks CA, two models reached statistical significance, one of the models included, as in the case of the risk of CP, all the investigated items: GM exam at 12 weeks CA (absent fidgety), AT 12 scarf sign(abnormal), AT 12 popliteal angle (abnormal), AT 12 axial tone (abnormal) and AT 12 synthesis (non-optimal) (*p* = 0.001; model 5), the odds ratio (Exp(B)) = 3.191, IC95 = 1.907–5.341.

The odds ratios for the predictors of independent walking can be viewed in Table 11.

**Figure 8:**
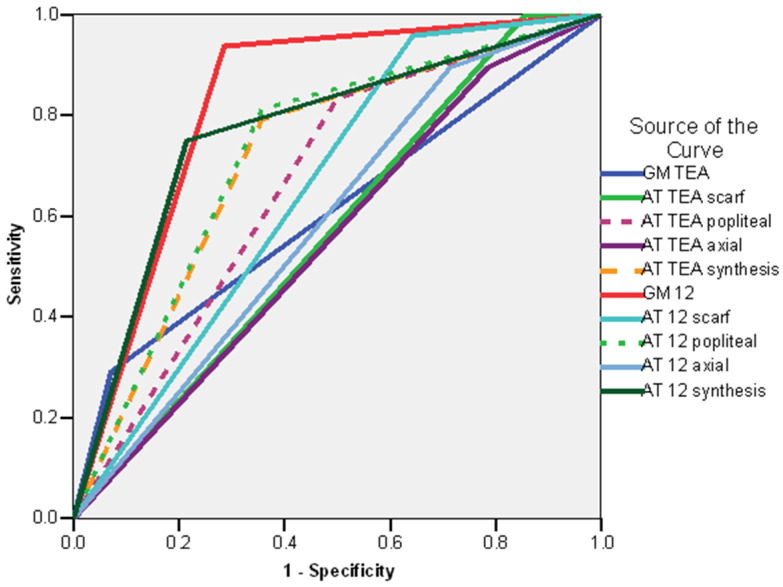
ROC curve—delayed/absent walking—preterm infants. Legend: AT TEA—Amiel Tison exam at Term Equivalent Age; AT 12—Amiel-Tison exam at 12 weeks corrected age; GM TEA—general movements assessment at Term Equivalent Age. GM 12 general movements assessment at 12 weeks corrected age.

The ROC curve (Figure 8) revealed the following factors to be good predictors of delayed/absent walking (AUC > 0.600): GM pattern at 12 weeks CA (absent fidgety) (AUC = 0.826; CI 95%: 0.678–0.974; *p* < 0.001), synthesis of the AT exam at 12 weeks CA (AUC = 0.768; CI 95%: 0.624–0.912; *p* < 0.002) and AT exam at 12 weeks CA—popliteal angle (AUC = 0.728; CI 95%: 0.566–0.890; *p* < 0.010), the synthesis of the AT exam at TEA (AUC = 0.717; CI 95%: 0.555–0.880; *p* = 0.014), and the AT exam at TEA—popliteal angle (AUC = 0.667; CI 95%: 0.493–0.841; *p* < 0.050).

The tables for the logistic regression models for delayed/absent walking can be consulted in Appendix A (Table A11 and Table A12). No model has reached statistical significance.

As in the case of the analysis of the whole group, due to the small sample and the small number of patients with CP, these models are exploratory and prone to overfitting, and more studies on larger samples of patients are needed before we can attempt to generalize the findings.

### 3.6. Predictive Values and Regression Models—Synthesis and Comparisons

The following tables are a synthesis of the predictive values of the individual items/examinations (Table 12) and the binary logistic regression models (Table 13).

The absence of fidgety movements at 12 weeks CA was the strongest individual predictor for all the outcomes, followed by the synthesis of the Amiel-Tison examinations at TEA and 12 weeks CA (predictor for all the outcomes except delayed sitting in the group of preterm infants). For the risk of CP, an abnormal popliteal angle at 12 weeks CA was a good predictor in the case of the whole group and subgroup of preterm infants, while an abnormal scarf sign was a good predictor for CP in the preterm infants group. Abnormal popliteal angles both at TEA and 12 weeks CA have also been identified as good predictors for delayed/absent walking.

An atypical GM pattern at TEA (PR or CS) was found to be predictive for all the outcomes except delayed sitting in the whole group (Table 13).

The combination of features of the GMA and AT examinations at 12 weeks CA is a predictor for the diagnosis of CP, both in the whole group and the subgroup of preterm infants, and for delayed sitting in the subgroup of preterm infants. The absence of fidgety movements has also been the strongest individual predictor in the logistic regression models (Table 13).

Table 14, Table 15 and Table 16 present a synthesis of the sensitivity, specificity, positive predictive value, negative predictive value, and AUC for certain predictors for the risk of CP, delayed sitting, and delayed/absent walking in the case of the whole group and the subgroup of preterm infants.

## 4. Discussion

### 4.1. Interpretation of Main Results

Our research showed that in a population of preterm infants and infants with different congenital conditions, the neurologic examinations performed at TEA and 12 weeks CA had a good predictive value in the early identification of infants at risk of CP and gross motor delay. Combining the results of GMA and AT examinations at 12 weeks CA could improve the early detection of these pathologies.

Even if a couple of the regression models showed statistical significance, due to many limitations—see the section below for more details on the limitations of the study—the results should be regarded with caution, and the reader is advised not to over-interpret the odds ratios—this is one of the reasons why the tables showing the odds ratios have been moved to the Supplementary Materials Section.

A good individual predictor for all the outcomes followed has been the absence of the fidgety movements at 12 weeks CA. Indeed, the GM evaluation has the greatest predictive value for the risk of CP [4] during the first few months of life and has been a topic of many studies showing its capacity to early identify the infants at risk of CP [17,24], as well as other outcomes [37] and has proven its value both in preterm infants and infants with other neurologic or genetic conditions [38]. We were surprised by the fact that we did not find a predictive value for the GM evaluation at TEA, but when separating the outcomes as normal (writhing pattern) and abnormal (PR and CS), the GM evaluation at term is a predictor for all the outcomes in the logistic regression model. An explanation could be that CS, but especially PR observed at TEA is known to develop into either the absence or presence of fidgety movements, while the normal pattern at term is always followed by the occurrence of fidgety movements [22]. This is probably why the GM exam at 12 weeks CA is a better predictor of outcomes than the GM exam at TEA.

Regarding the predictive values of the synthesis of the AT exam at TEA and 12 weeks CA, these have been good predictors for all the outcomes, except for delayed sitting in subgroup of preterm infants. Items involving the passive tone, especially popliteal angle at 12 weeks CA, were also predictors of the risk of CP, delayed sitting, and delayed/absent walking. The explanation of this finding is that in the category of preterm infants that represented the majority of the study sample, the type of CP observed is usually spastic, particularly involving the legs [4,39,40], and abnormalities of the tone of the legs are the first signs that appear in these situations [41].

In a study investigating the inter-observer reliability of the items of the AT exam, even if the reliability was good for both the synthesis and the individual items, the lowest reliability has been found for the pull-to-sit maneuver [14]. This could be an explanation for the lowest predictive value of this item in our group for almost all the outcomes investigated.

We identified logistic regression models that reached statistical significance for the risk of CP, both in the whole group and in the subgroup of preterm infants and models predictive of delayed sitting in the subgroup of preterm infants. All the models combined the findings of the examinations at 12 weeks CA. The absence of fidgety movements and an abnormal passive tone (abnormal scarf sign and abnormal popliteal angle) are part of all the models. In the case of the preterm infant subgroup, an abnormal synthesis of the AT exam at 12 weeks is also a part of the model. The explanation for these findings resides in the pathophysiologic context of the CP. The lesions that determine CP, especially in former preterm infants, occur at the level of the motor pathways [42]. The ability of the GM patterns, and especially of (absent) fidgety movements, to identify the patients at risk of CP could be explained by the relation between the GM patterns and the integrity of the motor pathways. Thus, even if GMs originate subcortically [17], the variability and modulation of these movements occurs at a cortical level [17,20,21], giving them a normal appearance (pattern). In the case of patients with CP, the interruption of the motor pathways and the destruction of the sub-plate neurons—the lesions that are responsible for the disease—also result in a lack of modulation of GMs and abnormal GM pattern results [20,21]. According to previous studies, fidgety movements could also represent a fine-tuning of the proprioceptive system [17,43]. Lesions within the motor pathways also lead to a release of the gamma moto-neurons from the cortical inhibition, which leads to hypertonicity and exaggerated monosynaptic reflexes [41]. Thus, these signs (increased passive tone of the arms and legs and absence of fidgety movements) are all early markers of the lesions at the levels of the motor pathways and abnormal proprioception. Their identification is usually an early warning sign for the risk of CP [9,12,17]. The absence of the synthesis of the AT examination from the predictive models for all the groups could be explained at least in part by the results of the AT examination at 12 weeks CA in patients with congenital neurologic conditions and syndromes (see Supplementary Material S1). The synthesis of the AT examination is abnormal in half of the patients with CP and half of the patients without CP. Even if the number of patients in this subgroup is small and no reliable statistical analysis can be performed because of this, one should be aware that an abnormal/non-optimal neurologic exam in this group may not solely be due to tone abnormalities, since the involvement of the central nervous system can occur in different ways in these conditions [9,13]. It is again to be mentioned that the models are just exploratory and should not be over-interpreted.

### 4.2. Comparison with Previous Studies

It is already known from the medical literature [17,18] that there is a good correlation between the GM movement patterns (writhing, poor repertoire and cramped–synchronized) at TEA and the GM movement patterns identified after 9 weeks of CA (based on the presence or absence of fidgety movements). This was also the case in our research (see Table 4), and, as already demonstrated, the correlation is stronger for normal results of the examination [22]—all the patients with normal GM patterns at TEA presented with fidgety movements. We found, though, a statistically significant correlation between the GM patterns at TEA and the ATNAT results (Table 7). The correlation was better for the normal/optimal results. This good correlation between the results of the AT exam and the GM patterns has also been found at 12 weeks of CA. These good correlations have also been found by other groups [25,26] and in our opinion, could be explained at least in part by the fact that both examinations, even if they have different physiological backgrounds, are based on the concept of optimality [11,12,13,18]. The correlation has been observed not only for the synthesis of the AT examinations, but also for the selected items.

Integration of two examination techniques in order to increase the predictive power for the neuro-motor outcomes has been a topic of research before—Romeo and co-workers demonstrated that integrating the findings of GM assessment and HINE offers a better prediction for CP than either of the examinations alone [27]. The novelty of our approach, even if the small number of subjects requires the results to be regarded with caution, is to integrate findings of the two examinations in binary logistic regression models.

### 4.3. Limitations, Strong Points, and Future Directions

We are aware of the important limitations and weak points of the study. The most important one is the small sample studied. This is why we advise caution in interpreting the results, and we consider that a validation study in a larger and more homogenous group is needed. The small number of cases results in a limitation of the validity and value of the logistic regression models. With only seventy infants overall and fewer than twenty cases of cerebral palsy, the logistic regression models are inevitably underpowered and vulnerable to unstable estimates. This affects the reliability of the wide confidence intervals observed in some models.

Another limitation is represented by the inhomogeneous sample, with preterm infants and infants with congenital neurologic conditions being analyzed together. Although we considered this to be a limitation, we consider that including term neonates and neonates with congenital conditions in the group could increase the value of the findings, showing the predictive value of the tests in children with different pathologies. Of course, the groups needed also to be analyzed separately. The small size of the group of infants with congenital conditions did not allow for a correct statistical analysis, but we analyzed the group of preterm infants separately. Another weak point could be the high incidence of CP both in the whole group (21.42%) and in the subgroup of former preterm infants (17.74%). Even if it is known that the incidence of CP is higher in low and middle-income countries [39] than in high-income ones [40], the incidence in our sample is much higher than that reported—21.42% vs. 3.4‰ [39,40]—which is also true for the preterm infants subgroup. This could represent an involuntary selection bias, because patients with neurological problems have the tendency to attend follow-up visits more frequently than those perceived as having normal development [44]. The high incidence of CP and potential selection bias limit the generalizability of predictive values, and the models should be considered exploratory. Even if the CP has been classified at diagnosis, due to the small number of cases in each GMFCS category, we did not perform an analysis stratified on each subtype of CP and GMFCS grade. 

The rate of loss from the follow-up was 9.09% of the whole group, with 0% in the term neonates and 10.14% in the preterm infants group. We decided from the beginning not to include in the study patients who did not complete the full program of visits, and this fact should lead to greater caution when it comes to over-interpreting the results. We believe that the high return rate in the term neonates is due to the severity of their conditions and the ability of our team to counsel them appropriately. In the case of the preterm infants, though, there has been, as expected, a drop-off rate from the program, but this has not been so high, although this fact is necessary to mention and certainly is a factor limiting the value of the findings.

We identified a number of confounding factors that could be considered in the interpretation of the data. Although the AT and GMA examinations were performed by different persons, a risk of interpretative bias could be present, because the persons performing the examinations were aware of the history and the results of the imaging studies of the patients. Also, both the examiners were trained in both methods—AIT had 20 years of experience performing AT examinations and received formal training and certification for GMA in 2023, while ARB had 10 years of experience in AT exam and also received training and certification in GMA in 2023. We did not cross-check for the inter-observer reliability, but GMA examination is known to have a very good inter-observer concordance [17,18,23,24]. This has also been the case for the AT examination (see above for the discussion in the Introduction; also see reference [14]). In our group, the AT examinations were performed by AIT and the GMA exams were scored by ARB. The interpretation bias could, however, result from the fact that both examiners were trained in the two methods, and this could induce an involuntary interpretation of the results based on observing other signs/items from the other technique, more on the side of the examiner that performed the AT examinations.

Our cohort of patients consisted of term and preterm neonates. Even if we analyzed them together, as stated above, the neonates with genetic conditions/CNS malformations are different from the former preterm infants regarding the type of lesions they have and the risk of CP [42,45,46]. This could be a confounding factor and this is why we attempted to separate the group of preterm infants from the whole group—they are more homogenous regarding the pathology and the type of CP encountered [42].

The gestational age distribution of our cohort is also slightly different from the studies that compared AT with GM examinations [25,26]. Our cohort has 4/62 patients with gestational ages of less than 30 weeks, while the cohort of Paro-Panjan and co-workers had 8/45 patients with GA of less than 30 weeks—in both cohorts, about half of the patients had a GA between 33 and 36 weeks. Thus, our cohort has a lower risk of adverse outcomes, but the data about the AT and GA examinations were comparable, because in both cases, the examinations were performed at TEA and 3 months CA/12 weeks CA in our study. Regarding the duration of follow-up, the previously cited cohorts surveyed the patients until 12–15 months corrected age [25,26] while our sample was followed until 24 months corrected age—it is known that a longer surveillance period is better because it allows more infants with anomalies to be identified [29,44]. It is known that the risk of CP and motor deficits is higher at lower gestational ages [39,40]. Accordingly, there could be two kinds of confounders—the higher gestational age in our sample, which would result in a lower number of infants at risk of CP and motor delays, and the longer interval of follow-up in our research, which could help to identify more infants with abnormalities than in the case of a shorter follow-up period up of 12–15 months (as in the previous cohorts). 

Regarding the logistic regression models, given the sample size and event counts, these multivariable models are exploratory and prone to overfitting. This statement should be strongly considered when analyzing the predictive value of the models and the general value of the study.

There are, though, in our opinion, a couple of strong points of this research. The first one is represented by the methods used to avoid interpretation bias [32] (see the Methods Section). The persons performing the GM and AT examinations were different and were not aware of the results of each other’s evaluation, and the person responsible for the statistics was form another center, with no medical background, and received only the anonymized database together with the research questions. Also, a strong point of the research is its examination of the same subjects by the two methods (GMA and AT), making the comparison of the results more reliable.

Another strong point of this research is the attempt to use a binary logistic regression model to provide an objective estimate of the predictive value of adding the two examinations together at TEA and 12 weeks CA. The use of binary logistic regression models required a simplification of the coding of the values of the exam in normal and abnormal cases [35,36] in order to obtain binary outcomes and to simplify the analysis.

In future, it is necessary to replicate this research in a multicenter study, with a larger number of patients and a more homogenous and larger gestational age distribution, in order to increase the value of the findings. Also, it would be worth investigating whether the use of the two examinations could predict other outcomes (fine motor, language, cognitive, behavioral).

## 5. Conclusions

GMA examination, and especially the absence of fidgety movements at 12 weeks CA, represents the strongest individual predictor of the occurrence of CP and of delayed gross motor milestones in our sample of term and preterm infants. The results of Amiel-Tison neurologic assessment at term (ATNAT) and Amiel-Tison neurologic exam of the infant at 12 weeks CA are also good predictors for the occurrence of CP and delayed sitting and delayed/absent walking, both in the whole group and in the subgroup of preterm infants. There was a good correlation between the results of the GMA evaluations and Amiel-Tison examinations, both at TEA and 12 weeks CA. Combining the findings of the GM and AT examinations suggests an improvement in the predictive performance for the detection of CP in the whole group and CP and delayed sitting in the subgroup of former preterm infants. Based on our findings, using the two examination techniques could enhance early detection of the infants at risk of motor abnormalities and refer them to appropriate early intervention programs, although this result should be validated in larger and more homogenous cohorts of infants.

All the findings of our study (especially the logistic models) pertain to this specific heterogeneous sample. Due to homogeneity issues, the findings that could be generalized (although with caution) are those based on the subgroup of preterm infants. Caution should, though, be used when interpreting the results of this research due to sample composition and size limitations, and larger studies are needed before robust conclusions can be drawn.

## Figures and Tables

**Table 1:** Protocol of the neurological examinations.

TEA	12 Weeks CA	7 Months CA	12 Months CA	18 Months CA	24 Months CA
GMA	GMA	-	-	-	-
ATNAT	Amiel-Tison 0–6 years	Amiel-Tison 0–6 years	Amiel-Tison 0–6 years	Amiel-Tison 0–6 years	Amiel-Tison 0–6 years

Legend: TEA—term equivalent age. CA—corrected age. ATNAT = Amiel Tison Neurological Assessment at term.

**Table 2:** Scores at different items in ATNAT and AT exam of the infant [9,12,13].

	Optimal	Non-Optimal/Abnormal
**Examination at TEA**		
Axial tone—raise to sit and reverse maneuver	Easy to obtain—in axis	Raise to sit—contraction of muscles, but no passage/no response Reverse—back-to-back—excessive response/no response
Upper limbs—scarf sign	The elbow does not touch the midline	The elbow can be displaced below the midline
Lower limbs—popliteal angle	70–90°	>100°
**Examination at 12 weeks CA**		
Axial tone—head control	Maintenance of the head in the axis of the boy for at least 15 s	- Maintenance for <15 s - Does not maintain the head
Upper limbs—scarf sign	The elbow can be displaced below the midline but not over the opposite mammary line	The elbow does not touch the midline—hypertonia The elbow can be displaced all the way to the opposite shoulder, or there may be no resistance at all—hypotonia
Lower limbs—popliteal angle	≥90°	<90°

**Table 3:** Correlation between the results of the GMA evaluation at TEA and 12 weeks of CA.

12 Weeks CA GM	GM TEA				Chi-Square Test Likelihood Ratio *p* Value
	CS (n = 17)	PR (n = 37)	Normal (n = 16)	All (n = 70)	
Absent fidgety	6 (35.3%)	9 (24.3%)	0 (0.0%)	15 (21.4%)	<0.008
Present fidgety	11 (64.7%)	28 (75.7%)	16 (100.0%)	55 (78.6%)	

Legend: CA—corrected age; CS—cramped–synchronized; GM—general movements; PR—poor repertoire; n—number of cases.

**Table 4:** Risk of CP—GMA exam and Amiel-Tison synthesis and items at TEA and 12 weeks CA—whole group.

	CP (n = 15)	Non-CP (n = 55)	Chi-Square Test Likelihood Ratio *p*
General Movements			
GM TEA			<0.103
CS	6 (40.0%)	11 (20.0%)	
PC	8 (53.3%)	29 (52.7%)	
Normal	1 (6.7%)	15 (27.3%)	
GM 12-week CA			<0.001
Absent	12 (80.0%)	3 (5.5%)	
fidgety	3 (20.0%)	52 (94.5%)	
Amiel-Tison Examinations			
AT TEA scarf			<0.008
Hypertonia	2 (13.3%)	0 (0.0%)	
Hypotonia	1 (6.7%)	0 (0.0%)	
Normal	12 (80.0%)	55 (100.0%)	
AT TEA popliteal			<0.013
Hypotonia	1 (6.7%)	0 (0.0%)	
Hypertonia	7 (46.7%)	10 (18.2%)	
Normal	7 (46.7%)	45 (81.8%)	
AT TEA axial			<0.014
Hypotonia	4 (26.7%)	7 (12.7%)	
Hypertonia	2 (12.3%)	0 (0.0%)	
Normal	6 (60.0%)	48 (87.3%)	
AT40 synthesis			<0.003
Optimal	5 (33.3%)	42 (76.4%)	
Non-optimal	10 (67.3%)	13 (23.6%)	
AT12 scarf			<0.001
Normal	9 (60.0%)	54 (98.2%)	
Abnormal	6 (40.0%)	1 (1.8%)	
AT12 popliteal			<0.001
Hypertonia	10 (66.7%)	11 (20.0%)	
Normal	5 (33.3%)	44 (80.0%)	
AT12 axial			<0.035
Hypotonia	5 (33.3%)	7 (12.7%)	
Hypertonia	1 (6.7%)	0 (0.0%)	
Normal	9 (60.0%)	48 (87.3%)	
AT12 synthesis			<0.001
Optimal	3 (20.0%)	39 (70.9%)	
Non-optimal	12 (80.0%)	16 (29.1%)	

Legend: AT TEA—Amiel-Tison exam at Term Equivalent Age. AT 12—Amiel-Tison Exam at 12 weeks corrected age; CA—corrected age; CP—cerebral palsy; CS—cramped–synchronized; GM—general movements, PR—poor repertoire; n—number of cases.

**Table 5:** Risk of delayed sitting—GMA exam and Amiel-Tison exam synthesis and items at TEA and 12 weeks CA—whole group.

	Sitting Delayed (n = 15)	Sitting On Time (n = 55)	Chi-Square Test Likelihood Ratio *p*
General Movements			
GM 40			<0.668
Cs	5 (33.3%)	12 (21.8%)	
Pr	7 (46.7%)	30 (54.5%)	
Normal	3 (20.0%)	13 (23.6%)	
GM 12			<0.002
Absent	8 (53.3%)	7 (12.7%)	
Fidgety	7 (46.7%)	48 (87.3%)	
Amiel-Tison Examinations			
AT TEA scarf			<0.133
Hypertonia	1 (6.7%)	1 (1.8%)	
Hypotonia	1 (6.7%)	0 (0.0%)	
Normal	13 (86.7%)	44 (98.2%)	
AT TEA popliteal			<0.049
Hypotonia	1 (6.7%)	0 (0.0%)	
Hypertonia	6 (40.0%)	11 (20.0%)	
Normal	8 (53.3%)	44 (80.0%)	
AT TEA axial			<0.272
Hypotonia	4 (26.7%)	7 (12.7%)	
Hypertonia	1 (6.7%)	1 (1.8%)	
Normal	10 (66.7%)	47 (85.5%)	
AT40 synthesis			<0.058
Optimal	7 (46.7%)	40 (72.7%)	
Non-optimal	8 (53.3%)	15 (27.3%)	
AT12 scarf			<0.163
Normal	12 (80.0%)	51 (92.7%)	
Abnormal	3 (20.0%)	4 (7.3%)	
AT12 popliteal			<0.104
Hypertonia	7 (46.7%)	14 (25.5%)	
Normal	8 (53.3%)	41 (74.5%)	
AT12 axial			<0.035
Hypotonia	5 (33.3%)	7 (12.7%)	
Hypertonia	1 (6.7%)	0 (0.0%)	
Normal	9 (60.0%)	48 (87.3%)	
AT12 synthesis			<0.019
Optimal	5 (33.3%)	37 (67.3%)	
Non-optimal	10 (66.7%)	18 (32.7%)	

Legend: AT TEA—Amiel-Tison exam at Term Equivalent Age. AT 12—Amiel-Tison Exam at 12 weeks corrected age; CA—corrected age; CS—cramped–synchronized; GM—general movements, PR—poor repertoire; n—number of cases.

**Table 6:** Risk of delayed/absent walking—GMA exam and Amiel Tison exam synthesis and items at TEA and 12 weeks CA—whole group.

	Walking Delayed/Absent (n = 18)	Walking On Time (n = 52)	Chi-Square Test Likelihood Ratio *p*
General movements			
GM 40			<0.160
CS	7 (38.9%)	10 (19.2%)	
PR	9 (50.0%)	28 (53.8%)	
Normal	2 (11.1%)	14 (26.9%)	
GM 12			<0.001
Absent	12 (66.7%)	3 (5.8%)	
Fidgety	6 (33.3%)	49 (94.2%)	
Amiel-Tison’s examinations			
AT TEA scarf			<0.014
Hypertonia	2 (11.1%)	0 (0.0%)	
Hypotonia	1 (5.6%)	0 (0.0%)	
Normal	15 (100.0%)	52 (100.0%)	
AT TEA popliteal			<0.015
Hypotonia	1 (5.6%)	0 (0.0%)	
Hypertonia	8 (44.4%)	9 (17.3%)	
Normal	9 (50.0%)	43 (82.7%)	
AT TEA axial			<0.035
Hypotonia	4 (22.2%)	7 (13.5%)	
Hypertonia	2 (11.1%)	0 (0.0%)	
Normal	12 (66.7%)	45 (86.5%)	
AT40 synthesis			<0.004
Optimal	7 (38.9%)	40 (76.9%)	
Non-optimal	11 (61.1%)	12 (23.1%)	
AT12 scarf			<0.010
Normal	13 (72.2%)	50 (96.2%)	
Abnormal	5 (27.8%)	2 (3.8%)	
AT12 popliteal			<0.002
Hypertonia	11 (61.1%)	10 (19.2%)	
Normal	7 (38.9%)	42 (80.8%)	
AT12 axial			<0.027
Hypotonia	6 (33.3%)	6 (11.5%)	
Hypertonia	1 (5.6%)	0 (0.0%)	
Normal	11 (84.7%)	46 (88.5%)	
AT12 synthesis			<0.001
Optimal	4 (22.2%)	38 (73.1%)	
Non-optimal	14 (77.8%)	14 (26.9%)	

Legend: AT TEA—Amiel-Tison exam at Term Equivalent Age. AT 12—Amiel-Tison Exam at 12 weeks corrected age; CA—corrected age; CS—cramped–synchronized; GM—general movements, PR—poor repertoire; n—number of cases.

**Table 7:** Correlation between AT exam and GM patterns at TEA.

AT TEA Results	GM Patterns				Chi-Square Test Likelihood Ratio *p*
	CS (n = 17)	PR (n = 37)	Normal (n = 16)	All (n = 70)	
AT TEA scarf					<0.366
Hypertonia	1 (5.9%)	1 (2.7%)	0 (0.0%)	2 (2.9%)	
Hypotonia	1 (5.9%)	0 (0.0%)	0 (0.0%)	1 (1.4%)	
Normal	15 (88.2%)	36 (97.3%)	16 (100.0%)	67 (95.7%)	
AT TEA popliteal					<0.001
Hypotonia	0 (0.0%)	1 (2.7%)	0 (0.0%)	1 (1.4%)	
Hypertonia	10 (58.8%)	7 (18.9%)	0 (0.0%)	17 (24.3%)	
Normal	7 (41.2%)	29 (78.4%)	16 (100.0%)	52 (74.3%)	
AT TEA axial					<0.015
Hypotonia	3 (17.6%)	8 (21.6%)	0 (0.0%)	11 (15.7%)	
Hypertonia	2 (11.8%)	0 (0.0%)	0 (0.0%)	2 (2.9%)	
Normal	12 (70.6%)	29 (78.4%)	16 (100.0%)	57 (81.4%)	
ATTEA synthesis					<0.001
Optimal	5 (29.4%)	26 (70.3%)	16 (100.0%)	47 (67.1%)	
Non-optimal	12 (70.6%)	11 (29.7%)	0 (0.0%)	23 (32.9%)	

Legend: AT TEA—Amiel-Tison exam at Term Equivalent Age; CS—cramped–synchronized; GM—general movements, PR—poor repertoire; n—number of cases.

**Table 8:** Correlation between AT exam and GM patterns at 12 weeks CA.

AT12	GM 12			Chi-Square Test Likelihood Ratio *p*
	Absent (n = 15)	Fidgety (n = 55)	All (n = 70)	
AT12 scarf				<0.001
Normal	9 (60.0%)	54 (98.2%)	63 (90.0%)	
Abnormal	6 (40.0%)	1 (1.8%)	7 (10.0%)	
AT12 popliteal				<0.001
Hypertonia	12 (80.0%)	9 (16.4%)	21 (30.0%)	
Normal	3 (20.0%)	46 (83.6%)	49 (70.0%)	
AT12 AX				<0.035
Hypotonia	5 (33.3%)	7 (12.7%)	12 (17.1%)	
Hypertonia	1 (6.7%)	0 (0.0%)	1 (1.4%)	
Normal	9 (60.0%)	48 (87.3%)	57 (81.4%)	
AT12 synthesis				<0.001
Optimal	2 (13.3%)	40 (72.7%)	42 (60.0%)	
Non-optimal	13 (86.7%)	14 (27.3%)	28 (40.0%)	

Legend: AT 12—Amiel-Tison Exam at 12 weeks corrected age; CA—corrected age; n—number of cases.

**Table 9:** Risk of CP. Subgroup of preterm infants (odds ratio).

	CP (n = 11)	Non-CP (n = 51)	Chi-Square Test *p*	OR	CI 95%
GM TEA			<0.034	-	-
Abnormal	11 (100.0%)	36 (70.6%)			
Normal	0 (0.0%)	15 (29.4%)			
AT TEA scarf			<0.029	6.67	3.65–12.18
Abnormal	2 (18.2%)	0 (0.0%)			
Normal	9 (81.8%)	51 (100.0%)			
AT TEA popliteal			<0.018	5.60	1.40–22.44
Abnormal	6 (54.5%)	9 (17.6%)			
Normal	5 (45.5%)	42 (82.4%)			
AT TEA axial			<0.142	3.45	0.69–17.37
Abnormal	3 (27.3%)	5 (9.8%)			
Normal	8 (72.7%)	46 (90.2%)			
AT TEA synthesis			<0.002	9.70	2.20–42.82
Non-optimal	8 (72.7%)	11 (21.6%)			
Optimal	3 (27.3%)	40 (78.4%)			
GM 12			<0.001	160.0	15.05–1700.5
Absent	10 (90.9%)	3 (5.9%)			
Fidgety	1 (9.1%)	48 (94.1%)			
AT 12 scarf			<0.001	60.0	5.97–603.25
Abnormal	6 (54.5%)	1 (2.0%)			
Normal	5 (45.5%)	50 (98.0%)			
AT 12 popliteal			<0.001	10.93	2.45–48.81
Abnormal	8 (72.7%)	10 (19.6%)			
Normal	3 (27.3%)	41 (80.4%)			
AT 12 axial			<0.191	2.81	0.58–13.61
Abnormal	3 (27.3%)	6 (11.8%)			
Normal	8 (72.7%)	45 (88.2%)			
AT 12 synthesis			<0.001	11.89	2.28–61.99
Non-optimal	9 (81.8%)	14 (27.5%)			
Optimal	2 (18.2%)	37 (72.5%)			

Legend: AT TEA—Amiel-Tison exam at Term Equivalent Age; AT 12—Amiel-Tison exam at 12 weeks corrected age; CI—confidence interval; GM TEA—general movements assessment at Term Equivalent Age. GM 12 general movements assessment at 12 weeks corrected Age. OR—Odds ratio. n—number of cases

**Table 10:** Risk of delayed sitting. Odds Ratios—subgroup of preterm infants.

	Delayed (n = 11)	Sitting on Time (n = 51)	Chi-Square Test *p*	OR	CI 95%
GM TEA			<0.469	1.54	0.29–8.07
Abnormal	9 (81.8%)	38 (74.5%)			
Normal	2 (18.2%)	13 (25.5%)			
AT TEA scarf			<0.326	5.00	0.29–86.76
Abnormal	1 (9.1%)	1 (2.0%)			
Normal	10 (90.9%)	50 (98.0%)			
AT TEA popliteal			<0.081	3.42	0.87–13.49
Abnormal	5 (45.5%)	10 (19.6%)			
Normal	6 (54.5%)	41 (80.4%)			
AT TEA axial			<0.435	1.67	0.29–9.62
Abnormal	2 (18.2%)	6 (11.8%)			
Normal	9 (81.8%)	45 (88.2%)			
AT TEA synthesis			<0.066	3.51	0.92–13.44
Non-optimal	6 (54.5%)	13 (25.5%)			
Optimal	5 (45.5%)	38 (74.5%)			
GM 12			<0.007	7.54	1.81–31.52
Absent fidgety	6 (54.5%)	7 (13.7%)			
Fidgety	5 (45.5%)	44 (87.3%)			
AT 12 scarf			<0.099	4.41	0.83–23.50
Abnormal	3 (27.3%)	4 (7.8%)			
Normal	8 (72.7%)	47 (92.2%)			
AT 12 popliteal			<0.168	2.44	0.64–9.34
Abnormal	5 (45.5%)	13 (25.5%)			
Normal	6 (54.5%)	38 (74.5%)			
AT 12 axial			<0.191	2.81	0.58–13.61
Abnormal	3 (27.3%)	6 (11.8%)			
Normal	8 (72.7%)	45 (88.2%)			
AT 12 synthesis			<0.048	3.83	0.98–14.97
Non-optimal	7 (63.6%)	16 (31.4%)			
Optimal	4 (36.4%)	35 (68.6%)			

Legend: AT TEA—Amiel-Tison exam at Term Equivalent Age; AT 12—Amiel-Tison exam at 12 weeks corrected age; CI—confidence interval; GM TEA—general movements assessment at Term Equivalent Age. GM 12 general movements assessment at 12 weeks corrected age; OR—odds ratio; n—number of cases.

**Table 11:** Odds ratios: independent walking—preterm infants.

	Walking Delayed (n = 14)	Walking on Time (n = 48)	Chi-Square Test *p*	OR	CI 95%
GM TEA			<0.084	5.35	0.64–44.91
Abnormal	13 (92.9%)	34 (70.8%)			
Normal	1 (7.1%)	14 (29.2%)			
AT TEA scarf			<0.048	5.00	3.01–8.29
Abnormal	2 (14.3%)	0 (0.0%)			
Normal	12 (85.7%)	48 (100.0%)			
AT TEA popliteal			<0.017	5.00	1.37–18.23
Abnormal	7 (50.0%)	8 (16.7%)			
Normal	7 (50.0%)	40 (83.3%)			
AT TEA axial			<0.253	2.35	0.48–11.35
Abnormal	3 (21.4%)	5 (10.4%)			
Normal	11(78.6%)	43 (89.6%)			
AT TEA synthesis			<0.003	6.84	1.87–25.01
Abnormal	9 (64.3%)	10 (20.8%)			
Normal	5 (35.7%)	38 (79.2%)			
GM 12			<0.001	37.50	7.23–194.54
Absent fidgety	10 (71.4%)	3 (6.3%)			
Present fidgety	4 (28.6%)	45 (93.8%)			
AT 12 scarf			<0.005	12.78	2.14–76.43
Abnormal	5 (35.7%)	2 (4.28%)			
Normal	9 (64.3%)	46 (94.8%)			
AT 12 popliteal			<0.002	7.80	2.10–28.96
Abnormal	9 (64.3%)	9 (18.8%)			
Normal	5 (35.7%)	39 (81.3%)			
AT 12 axial			<0.106	3.44	0.78–15.17
Abnormal	4 (28.6%)	5 (10.4%)			
Normal	10 (71.4%)	43 (89.6%)			
AT 12 synthesis			<0.001	11.00	2.62–46.15
Non-optimal	11(78.6%)	12 (25.0%)			
Optimal	3 (21.4%)	36 (75.0%)			

Legend: AT TEA—Amiel-Tison exam at Term Equivalent Age; AT 12—Amiel-Tison exam at 12 weeks corrected age; CI—confidence interval, GM TEA—general movements assessment at Term Equivalent Age. GM 12 general movements assessment at 12 weeks corrected age. OR—odds ratio. n—number of cases.

**Table 12:** Summative table—individual predictors—whole group and preterm infants group—see Supplementary Tables S2.1–S2.3 for the whole group and Table 9, Table 10 and Table 11 for the group of preterm infants for additional information.

	CP—Whole Group	CP—Preterm	Delayed Sitting—Whole Group	Delayed Sitting—Preterm	Delayed/Absent Walking—Whole Group	Delayed/Absent Walking—Preterm
GM TEA						
AT TEA scarf						
AT TEA popliteal						
AT TEA axial						
AT TEA synthesis						
GM 12 weeks CA						
AT 12 weeks CA scarf						
AT 12 weeks CA popliteal						
AT 12 weeks CA axial						
AT 12 weeks CA synthesis						

Legend: AT TEA—Amiel-Tison exam at Term Equivalent Age; AT 12—Amiel-Tison exam at 12 weeks corrected age; GM TEA—general movements assessment at Term Equivalent Age. GM 12 general movements assessment at 12 weeks corrected Age. 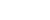 Not predictive; 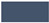 Predictive.

**Table 13:** Summative table. Logistic regression models—for supplementary information, see Table A1, Table A2, Table A3, Table A4, Table A5 and Table A6 for the whole group and Table A7, Table A8, Table A9, Table A10, Table A11 and Table A12 for the preterm infants group.

	CP—Whole Group	CP—Preterm	Delayed Sitting—Whole Group	Delayed Sitting—Preterm	Delayed/Absent Walking—Whole Group	Delayed/Absent Walking—Preterm
Exams at TEA	GM abnormal	GM abnormal		GM abnormal	GM abnormal	GM abnormal
Exams at 12 weeks CA	GM 12—absent fidgety Model 3: absent Fidgety + abnormal scarf + abnormal popliteal	GM 12—absent fidgety Model 3 Absent fidgety + abnormal scarf + abnormal popliteal Model 5 Absent fidgety + abnormal scarf + abnormal popliteal + abnormal axial + abnormal synthesis	GM 12—absent fidgety	Model 3 Absent fidgety + abnormal scarf + abnormal popliteal Model 5 Absent fidgety + abnormal scarf + abnormal popliteal + abnormal axial + abnormal synthesis	GM 12—absent fidgety	GM 12—absent fidgety.

Legend: AT TEA—Amiel-Tison exam at Term Equivalent Age; AT 12—Amiel-Tison exam at 12 weeks corrected age.; GM TEA—general movements assessment at Term Equivalent Age. GM 12 general movements assessment at 12 weeks corrected age.

**Table 14:** Risk of CP—sensitivity, specificity, positive predictive value, negative predictive value, AUC.

	Sensitivity	Specificity	Positive Predictive Value	Negative Predictive Value	AUC
Whole group					
Absent fidgety 12 weeks CA	80%	94.55%	80%	94.55%	0.873
AT exam at TEA synthesis—non-optimal	66.66%	76.36%	43.47%	89.36%	0.715
AT exam at 12 weeks—synthesis—non-optimal	80%	70.90%	42.85%	92.85%	0.755
Preterm infants subgroup					
Absent fidgety 12 weeks CA	90.9%	94.11%	76.92%	97.95%	0.925
AT exam at TEA synthesis—non-optimal	72.72%	78.43%	42.1%	93.02%	0.756
AT exam at 12 weeks—synthesis—non-optimal	81.81%	72.54%	39.13%	94.87%	0.772

**Table 15:** Delayed sitting—sensitivity, specificity, positive predictive value, negative predictive value, AUC.

	Sensitivity	Specificity	Positive Predictive Value	Negative Predictive Value	AUC
Whole group					
Absent fidgety 12 weeks CA	53.3%	82.72%	53.3%	82.72%	0.804
AT exam at 12 weeks—synthesis—non-optimal	66.66%	67.27%	35.71%	88.09%	0.754
Preterm infants subgroup					
Absent fidgety 12 weeks CA	54.54%	86.27%	46.15%	89.79%	0.704
AT exam at 12 weeks—synthesis—non-optimal	63.63%	68.62%	30.43%	89.74%	0.661

**Table 16:** Delayed/absent walking—sensitivity, specificity, positive predictive value, negative predictive value, AUC.

	Sensitivity	Specificity	Positive Predictive Value	Negative Predictive Value	AUC
Whole group					
Absent fidgety 12 weeks CA	66.66%	94.23%	80%	89.09%	0.804
AT exam at TEA synthesis—non-optimal	61.11%	76.92%	47.82%	85.10%	0.690
AT exam at 12 weeks—synthesis—non-optimal	77.77%	73.07%	50%	90.47%	0.754
Preterm infants subgroup					
Absent fidgety 12 weeks CA—non-optimal	71.42%	93.75%	76.92%	91.83%	0.826
AT exam at TEA synthesis	64.28%	79.16%	47.36%	88.37%	0.717
AT exam at 12 weeks—synthesis—non-optimal	78.56%	75%	47.82%	92.3%	0.768

## Data Availability

The database of the study can be accessed upon request at the address adrian.toma@prof.utm.ro.

## Supplementary Materials

The following supporting information can be downloaded at https://www.mdpi.com/article/10.3390/life16010081/s1, Table S1. General characteristics of the non-preterm (term) infants group. Table S2.1. Odds ratios for the risk of cerebral palsy (CP)—GM and Amiel-Tison examinations—whole group. Table S2.2. Odds ratios for the risk of delayed sitting—whole group Table S2.3. Risk of delayed/absent walking (odds ratio)—whole group.

## Abbreviations

AT	Amiel-Tison
ATNAT	Amiel-Tison Neurological Assessment at Term
ATNA	Amiel-Tison Neurological Assessment
B	Beta coefficient—logistic regression models
AUC	Area under the curve
CA	Corrected age
CI	Confidence interval
CP	Cerebral palsy
CS	Cramped–synchronized
Df	Degrees of freedom—logistic regression models
Exp(B)	The odds ratio for the predictor variable it is associated with
GA	Gestational age
GM	General movements
GMA	General movements assessment
GMFCS	Gross Motor Function Classification System
HINE	The Hammersmith Infant Neurological Examination
NICU	Neonatal intensive care unit
OR	Odds ratio
PR	Poor repertoire.
ROC curve	Receiver Operating Characteristic curve
S.E.	Standard error—logistic regression models
Sig.	Significance—logistic regression models
TEA	Term equivalent age
VIF	Variance inflation factor

## Appendix A

**Table A1:** Binary logistic regression. Dependent variable CP. Independent variables GM TEA, AT TEA scarf, AT TEA popliteal, AT TEA axial, AT TEA synthesis. Whole group.

		B	S.E.	Wald	df	Sig.	Exp(B)	95.0% C.I. for EXP(B)	
								Lower	Upper
Variable(s) entered on step 1: GM TEA.									
	GM TEA (abnormal)	1.050	0.311	11.429	1	0.001	2.857	1.555	5.251
Variable(s) entered on step 2: ATTEA scarf									
	GM TEA (abnormal)	1.291	0.340	14.379	1	0.001	3.636	1.866	7.087
	ATTEA scarf (abnormal)	−22.494	23,205.42	0.000	1	0.999	0.000	0.000	Not computed
Variable(s) entered on step 3: AT TEA popliteal									
	GM TEA (abnormal)	1.609	0.447	12.951	1	0.001	5.000	2.081	12.013
	ATTEA scarf (abnormal)	−21.896	23,205.42	0.000	1	0.999	0.000	0.000	.
	AT TEA popliteal (abnormal)	−0.916	0.707	1.679	1	0.195	0.400	0.100	1.599
Variable(s) entered on step 4: AT TEA axial									
	GM TEA (abnormal)	1.785	0.490	13.252	1	0.001	5.960	2.279	15.581
	ATTEA scarf (abnormal)	−21.696	22,890.913	0.000	1	0.999	0.000	0.000	.
	AT TEA popliteal (abnormal)	−0.785	0.726	1.169	1	0.280	0.456	0.110	1.893
	AT TEA axial (abnormal)	−0.848	0.773	1.205	1	0.272	0.428	0.094	1.947
Variable(s) entered on step 5: AT TEA synthesis.									
	GM TEA (abnormal)	2.197	0.609	13.035	1	0.001	9.000	2.730	29.667
	ATTEA scarf (abnormal)	−24.416	19,894.349	0.000	1	0.999	0.000	0.000	.
	AT TEA popliteal (abnormal)	1.386	1.384	1.003	1	0.317	4.000	0.265	60.325
	AT TEA axial (abnormal)	0.981	1.291	0.577	1	0.447	2.667	0.212	33.486
	ATTEA synthesis (normal)	−3.178	1.644	3.736	1	0.053	0.042	0.002	1.046

Legend: AT TEA—Amiel-Tison exam at Term Equivalent Age. GMs—general movements. Not computed—the value could not be computed by the software—the model is not considered valid.

**Table A2:** Binary logistic regression. Dependent variable CP. Independent variables GM12 weeks CA, AT12 weeks scarf, AT12 weeks CA popliteal, AT12 weeks CA axial, AT12 weeks CA—synthesis. Whole group.

		B	S.E.	Wald	df	Sig.	Exp(B)	95.0% C.I. for EXP(B)	
								Lower	Upper
Variable(s) entered on step 1: GM12.									
	GM12 (fidgeting absent)	−1.386	0.645	4.612	1	0.032	0.250	0.071	0.886
Variable(s) entered on step 2: AT12 scarf.									
	GM12 (fidgeting absent)	−1.078	0.734	2.161	1	0.142	0.340	0.081	1.433
	AT12 scarf (abnormal)	−0.922	1.228	0.564	1	0.453	0.398	0.036	4.412
Variable(s) entered on step 3: AT12 popliteal.									
	GM12 (fidgeting absent)	−2.911	1.238	5.532	1	0.019	0.054	0.005	0.616
	AT12 scarf (abnormal)	−1.259	1.253	1.009	1	0.315	0.284	0.024	3.310
	AT12 popliteal (abnormal)	2.267	1.054	4.622	1	0.032	9.649	1.222	76.208
Variable(s) entered on step 4: AT12 axial									
	GM12 (fidgeting absent)	−3.430	1.331	6.639	1	0.010	0.032	0.002	0.440
	AT12 scarf (abnormal)	−0.826	1.305	0.400	1	0.527	0.438	0.034	5.653
	AT12 popliteal (abnormal)	2.223	1.058	4.419	1	0.036	9.237	1.162	73.417
	AT12 axial (abnormal)	0.872	0.778	1.256	1	0.262	2.391	0.521	10.985
Variable(s) entered on step 5: AT12 synthesis									
	GM12 (fidgeting absent)	−3.275	1.386	5.580	1	0.018	0.038	0.002	0.572
	AT12 scarf (abnormal)	−2.822	1.720	2.691	1	0.101	0.060	0.002	1.733
	AT12 popliteal (abnormal)	0.887	1.202	0.544	1	0.461	2.427	0.230	25.598
	AT12 axial (abnormal)	−1.389	1.469	0.894	1	0.344	0.249	0.014	4.438
	AT12 synthesis (abnormal)	2.815	1.519	3.436	1	0.064	16.699	0.851	327.783

Legend: AT 12—Amiel-Tison Exam at 12 weeks corrected age; CA—corrected age; GM 12—general movements—12 weeks corrected age.

**Table A3:** Binary logistic regression. Dependent variable: sitting delayed. Independent variables GM TEA, AT TEA scarf, AT TEA popliteal, AT TEA axial, AT TEA synthesis.

		B	S.E.	Wald	df	Sig.	Exp(B)	95.0% C.I. for EXP(B)	
								Lower	Upper
Variable(s) entered on step 1: GM TEA.									
	GM TEA (abnormal)	−0.489	0.214	5.241	1	0.022	0.613	0.404	0.932
Variable(s) entered on step 2: AT TEA SCARF.									
	GM TEA (abnormal)	−0.018	0.244	0.006	1	0.940	0.982	0.609	1.583
	AT TEA scarf (abnormal)	−0.470	0.118	16.005	1	0.001	0.625	0.496	0.787
Variable(s) entered on step 3: AT TEA POPLITEAL.									
	GM TEA (abnormal)	0.119	0.267	0.200	1	0.655	1.127	0.667	1.902
	AT TEA scarf (abnormal)	−0.231	0.183	1.602	1	0.206	0.794	0.555	1.135
	AT TEA popliteal (abnormal)	−0.377	0.243	2.405	1	0.121	0.686	0.426	1.105
Variable(s) entered on step 4: AT TEA AXIAL.									
	GM TEA (abnormal)	0.159	0.274	0.336	1	0.562	1.172	0.685	2.004
	AT TEA scarf (abnormal)	−0.103	0.243	0.180	1	0.672	0.902	0.561	1.452
	AT TEA popliteal (abnormal)	−0.346	0.248	1.956	1	0.162	0.707	0.436	1.149
	AT TEA axial (abnormal)	−0.198	0.252	0.620	1	0.431	0.820	0.501	1.344
Variable(s) entered on step 5: AT TEA synthesis.									
	GM TEA (abnormal)	0.176	0.281	0.392	1	0.531	1.192	0.687	2.068
	AT TEA scarf (abnormal)	−0.138	0.268	0.267	1	0.606	0.871	0.515	1.472
	AT TEA popliteal (abnormal)	−0.257	0.373	0.473	1	0.492	0.774	0.372	1.608
	AT TEA axial (abnormal)	−0.143	0.306	0.217	1	0.641	0.867	0.476	1.579
	AT TEA synthesis (normal)	−0.127	0.402	0.100	1	0.751	0.880	0.400	1.936

Legend: AT TEA—Amiel-Tison exam at Term Equivalent Age. GMs—general movements.

**Table A4:** Binary logistic regression. Dependent variable: sitting delayed. Independent variables GM 12, AT 12 scarf, AT 12 popliteal, AT 12 axial, AT 12 synthesis.

		B	S.E.	Wald	df	Sig.	Exp(B)	95.0% C.I. for EXP(B)	
								Lower	Upper
Variable(s) entered on step 1: GM12.									
	GM 12 (absent)	−0.288	0.764	0.142	1	0.706	0.750	0.168	3.351
Variable(s) entered on step 2: AT12MS.									
	GM 12 (absent)	0.409	0.901	0.206	1	0.650	1.506	0.258	8.805
	AT 12 scarf (abnormal)	0.288	0.764	0.142	1	0.706	1.333	0.298	5.957
Variable(s) entered on step 3: AT12MI.									
	GM 12 (absent)	0.456	0.918	0.247	1	0.619	1.578	0.261	9.541
	AT 12 scarf (abnormal)	−0.409	0.901	0.206	1	0.650	0.664	0.114	3.883
	AT 12 popliteal (abnormal)	0.815	0.543	2.258	1	0.133	2.260	0.780	6.548
Variable(s) entered on step 4: AT12AX.									
	GM12 (absent)	0.288	0.764	0.142	1	0.706	1.333	0.298	5.957
	AT 12 scarf (abnormal)	−0.456	0.918	0.247	1	0.619	0.634	0.105	3.831
	AT 12 popliteal (abnormal)	0.870	0.578	2.268	1	0.132	2.387	0.769	7.407
	AT 12 axial (abnormal)	−0.171	0.605	0.079	1	0.778	0.843	0.258	2.760
Variable(s) entered on step 5: AT12 synthesis.									
	GM 12 (absent)	1.000	0.496	4.060	1	0.044	2.717	1.028	7.184
	AT 12 scarf (abnormal)	0.165	1.267	0.017	1	0.897	1.179	0.098	14.124
	AT 12 popliteal (abnormal)	−0.257	0.715	0.130	1	0.719	0.773	0.191	3.137
	AT 12 axial (abnormal)	0.432	0.501	0.742	1	0.389	1.540	0.577	4.112
	AT12 synthesis(abnormal)	−0.296	0.792	0.139	1	0.709	0.744	0.157	3.515

Legend: AT 12—Amiel-Tison Exam at 12 weeks corrected age; CA—corrected age; GM 12—general movements—12 weeks corrected age.

**Table A5:** Binary logistic regression. Dependent variable: delayed/absent walking. Independent variables GM TEA, AT TEA scarf, AT TEA popliteal, AT TEA axial, AT TEA synthesis.

		B	S.E.	Wald	df	Sig.	Exp(B)	95.0% C.I. for EXP(B)	
								Lower	Upper
Variable(s) entered on step 1: GM TEA.									
	GM TEA (abnormal)	0.865	0.298	8.424	1	0.004	2.375	1.324	4.259
Variable(s) entered on step 2: AT TEA scarf.									
	GM TEA (abnormal)	1.073	0.321	11.145	1	0.001	2.923	1.557	5.487
	AT TEA scarf (abnormal)	−22.276	23,205.42	0.000	1	0.999	0.000	0.000	Not computed
Variable(s) entered on step 3: AT TEA popliteal.									
	GM TEA (abnormal)	1.421	0.421	110392	1	0.001	4.143	1.815	9.457
	AT TEA scarf (abnormal)	−21.608	23,205.42	0.000	1	0.999	0.000	0.000	Not computed
	AT TEA popliteal (abnormal)	−1.016	0.675	2.268	1	0.132	0.362	0.097	1.358
Variable(s) entered on step 4: AT TEA axial.									
	GM TEA (abnormal)	1.515	0.451	11.274	1	0.001	4.551	1.879	11.023
	AT TEA scarf (abnormal)	−21.466	23,091.39	0.000	1	0.999	0.000	0.000	.
	AT TEA popliteal (abnormal)	−0.938	0.687	1.866	1	0.172	0.391	0.102	1.504
	AT TEA axial (abnormal)	−0.500	0.759	0.435	1	0.510	0.606	0.137	2.684
Variable(s) entered on step 5: AT TEA synthesis.									
	GM TEA (abnormal)	1.872	0.537	12.146	1	0.001	6.500	2.269	18.624
	AT TEA scarf (abnormal)	−24.454	19,564.15	0.000	1	0.999	0.000	0.000	Not computed
	AT TEA popliteal (abnormal)	1.386	1.384	1.003	1	0.317	4.000	0.265	60.325
	AT TEA axial (abnormal)	1.386	1.285	1.165	1	0.280	4.000	0.323	49.596
	AT TEA synthesis (normal)	−30.258	1.614	4.075	1	0.044	0.038	0.002	0.910

Legend: AT TEA—Amiel-Tison exam at Term Equivalent Age. GMs—general movements. Not computed—the value could not be computed by the software—the model is not considered valid.

**Table A6:** Binary logistic regression. Dependent variable CP. Independent variables GM 12, AT 12 scarf, AT 12 popliteal, AT 12 axial, and AT 12 synthesis.

		B	S.E.	Wald	df	Sig.	Exp(B)	95.0% C.I. for EXP(B)	
								Lower	Upper
Variable(s) entered on step 1: GM12.									
	GM 12 (absent)	−1.386	0.645	4.612	1	0.032	0.250	0.071	0.886
Variable(s) entered on step 2: AT12 scarf.									
	GM 12 (absent)	−1.540	0.815	3.571	1	0.050	0.214	0.043	1.059
	AT 12 scarf (abnormal)	0.360	1.095	0.108	1	0.742	1.434	0.168	12.270
Variable(s) entered on step 3: AT12 popliteal.									
	GM 12 (absent)	−2.640	1.083	5.944	1	0.015	0.071	0.009	0.596
	AT 12 scarf (abnormal)	0.127	1.101	0.013	1	0.908	1.136	0.131	9.817
	AT 12 popliteal (abnormal)	1.386	0.790	3.078	1	0.079	3.999	0.850	18.813
Variable(s) entered on step 4: AT12 axial									
	GM12 (absent)	−2.976	1.163	6.550	1	0.010	0.051	0.005	0.498
	AT 12 scarf (abnormal)	0.437	1.180	0.137	1	0.711	1.547	0.153	15.620
	AT 12 popliteal (abnormal)	1.343	0.792	2.874	1	0.090	3.832	0.811	18.110
	AT 12 axial (abnormal)	0.613	0.745	0.678	1	0.410	1.846	0.429	7.943
Variable(s) entered on step 5: AT12 synthesis.									
	GM 12 (absent)	1.000	0.496	4.060	1	0.044	2.717	1.028	7.184
	AT 12 scarf (abnormal)	0.165	1.267	0.017	1	0.897	1.179	0.098	14.124
	AT 12 popliteal (abnormal)	−0.257	0.715	0.130	1	0.719	0.773	0.191	3.137
	AT 12 axial (abnormal)	0.432	0.501	0.742	1	0.389	1.540	0.577	4.112
	AT12 synthesis(abnormal)	−0.296	0.792	0.139	1	0.709	0.744	0.157	3.515

Legend: AT 12—Amiel-Tison Exam at 12 weeks corrected age; CA—corrected age; GM 12—general movements—12 weeks corrected age.

**Table A7:** Binary logistic regression. Dependent variable: CP. Independent variables: GM TEA, AT TEA scarf, AT TEA popliteal, AT TEA axial, AT TEA synthesis. Subgroup of preterm infants.

		B	S.E.	Wald	df	Sig.	Exp(B)	95.0% C.I. for EXP(B)	
								Lower	Upper
Variable(s) entered on step 1: GM TEA.									
	GM TEA (abnormal)	1.186	0.345	11.844	1	0.001	3.273	1.666	6.429
Variable(s) entered on step 2: AT TEA SCARF.									
	GM TEA (abnormal)	1.386	0.373	13.837	1	0.001	4.000	1.927	8.304
	AT TEA scarf (abnormal)	−22.589	28,420.72	0.000	1	0.999	0.000	0.000	Not computed
Variable(s) entered on step 3: AT TEA POPLITEAL.									
	GM TEA (abnormal)	1.686	0.487	11.998	1	0.001	5.400	2.080	14.022
	AT TEA scarf (abnormal)	−22.014	28,420.72	0.000	1	0.999	0.000	0.000	.
	AT TEA popliteal (abnormal)	−0.875	0.773	1.281	1	0.258	0.417	0.092	1.897
Variable(s) entered on step 4: AT TEA AXIAL.									
	GM TEA (abnormal)	1.749	0.511	11.713	1	0.001	5.749	2.112	15.655
	AT TEA scarf (abnormal)	−21.918	28,310.09	0.000	1	0.999	0.000	0.000	.
	AT TEA popliteal (abnormal)	−0.830	0.782	1.128	1	0.288	0.436	0.094	2.017
	AT TEA axial (abnormal)	−0.441	0.958	0.212	1	0.645	0.643	0.098	4.206
Variable(s) entered on step 5: AT TEA synthesis.									
	GM TEA (abnormal)	2.120	0.611	12.042	1	0.001	8.333	2.516	27.600
	AT TEA scarf (abnormal)	−41.066	31,492.697	0.000	1	0.999	0.000	0.000	.
	AT TEA popliteal (abnormal)	20.805	19,017.791	0.000	1	0.999	1 × 10^9^	0.000	.
	AT TEA axial (abnormal)	20.399	19,017.791	0.000	1	0.999	7 × 10^9^	0.000	.
	AT TEA synthesis (normal)	−22.520	19,017.791	0.000	1	0.999	0.000	0.000	.

Legend: AT TEA—Amiel-Tison exam at Term Equivalent Age. GMs—general movements. Not computed—the value could not be computed by the software—the model is not considered valid.

**Table A8:** Binary logistic regression. Dependent variable: CP. Independent variables: GM 12, AT 12 scarf, AT 12 popliteal, AT 12 axial, AT 12 synthesis. Subgroup of preterm infants.

		B	S.E.	Wald	df	Sig.	Exp(B)	95.0% C.I. for EXP(B)	
								Lower	Upper
Variable(s) entered on step 1: GM12.									
	GM 12 (absent)	−1.204	0.658	3.345	1	0.067	0.300	0.083	1.090
Variable(s) entered on step 2: AT12MS.									
	GM 12 (absent)	−0.756	0.775	0.951	1	0.330	0.470	0.103	2.146
	AT 12 scarf (abnormal)	−1.170	1.246	0.882	1	0.348	0.310	0.027	3.568
Variable(s) entered on step 3: AT12MI.									
	GM 12 (absent)	−2.377	1.238	3.689	1	0.055	0.093	0.008	1.050
	AT 12 scarf (abnormal)	−1.705	1.315	1.683	1	0.195	0.182	0.014	2.390
	AT 12 popliteal (abnormal)	2.276	1.073	4.498	1	0.034	9.736	1.188	79.763
Variable(s) entered on step 4: AT12AX.									
	GM12 (absent)	−3.275	1.390	5.551	1	0.018	0.038	0.002	0.577
	AT 12 scarf (abnormal)	−1.027	1.367	0.564	1	0.453	0.358	0.025	5.224
	AT 12 popliteal (abnormal)	2.335	1.112	4.409	1	0.036	10.330	1.168	91.343
	AT 12 axial (abnormal)	1.645	1.069	2.366	1	0.124	5.179	0.637	42.117
Variable(s) entered on step 5: AT12 synthesis.									
	GM 12 (absent)	−3.275	1.386	5.580	1	0.018	0.038	0.002	0.572
	AT 12 scarf (abnormal)	−2.822	1.720	2.691	1	0.101	0.060	0.002	1.733
	AT 12 popliteal (abnormal)	0.887	1.202	0.544	1	0.461	2.427	0.230	25.598
	AT 12 axial (abnormal)	−1.389	1.469	0.894	1	0.344	0.249	0.014	4.438
	AT12 synthesis(abnormal)	4.815	1.519	3.436	1	0.046	16.699	1.851	327.783

Legend: AT 12—Amiel-Tison Exam at 12 weeks corrected age; CA—corrected age; GM 12—general movements—12 weeks corrected age.

**Table A9:** Binary logistic regression. Dependent variable: sitting delayed. Independent variables: GM TEA, AT TEA scarf, AT TEA popliteal, AT TEA axial, AT TEA synthesis. Subgroup of preterm infants.

		B	S.E.	Wald	df	Sig.	Exp(B)	95.0% C.I. for EXP(B)	
								Lower	Upper
Variable(s) entered on step 1: GM TEA.									
	GM TEA (abnormal)	1.440	0.371	15.096	1	0.001	4.222	2.042	8.732
Variable(s) entered on step 2: AT TEA scarf.									
	GM TEA (abnormal)	1.531	0.390	15.428	1	0.001	4.625	2.154	9.931
	AT TEA scarf (abnormal)	−1.531	1.467	1.090	1	0.297	0.216	0.012	3.833
Variable(s) entered on step 3: AT TEA popliteal.									
	GM TEA (abnormal)	1.946	0.535	13.253	1	0.001	7.000	2.455	19.957
	AT TEA scarf (abnormal)	−0.811	1.537	0.279	1	0.598	0.444	0.022	9.032
	AT TEA popliteal (abnormal)	−1.135	0.804	1.992	1	0.158	0.321	0.066	1.555
Variable(s) entered on step 4: AT TEA axial.									
	GM TEA (abnormal)	1.956	0.550	12.640	1	0.001	7.069	2.405	20.776
	AT TEA scarf (abnormal)	−0.791	1.559	0.257	1	0.612	0.454	0.021	9.628
	AT TEA popliteal (abnormal)	−1.127	0.811	1.931	1	0.165	0.324	0.066	1.588
	AT TEA axial (abnormal)	−0.076	0.989	0.006	1	0.939	0.927	0.133	6.434
Variable(s) entered on step 5: AT TEA synthesis.									
	GM TEA (abnormal)	1.154	0.556	0.077	1	0.050	1.167	0.392	3.471
	AT TEA scarf (abnormal)	−0.138	0.268	0.267	1	0.606	0.871	0.515	1.472
	AT TEA popliteal (abnormal)	−0.257	0.373	0.473	1	0.492	0.774	0.372	1.608
	AT TEA axial (abnormal)	−0.143	0.306	0.217	1	0.641	0.867	0.476	1.579
	AT TEA synthesis (normal)	−0.127	0.402	0.100	1	0.751	0.880	0.400	1.936

Legend: AT TEA—Amiel-Tison exam at Term Equivalent Age. GMs—general movements.

**Table A10:** Binary logistic regression. Dependent variable: sitting delayed. Independent variables: GM 12, AT 12 scarf, AT 12 popliteal, AT 12 axial, AT 12 synthesis. Subgroup of preterm infants.

		B	S.E.	Wald	df	Sig.	Exp(B)	95.0% C.I. for EXP(B)	
								Lower	Upper
Variable(s) entered on step 1: GM12.									
	GM 12 (absent)	0.154	0.556	0.077	1	0.782	1.167	0.392	3.471
Variable(s) entered on step 2: AT12 scarf.									
	GM 12 (absent)	0.036	0.714	0.003	1	0.959	1.037	0.256	4.205
	AT 12 scarf (abnormal)	0.256	0.979	0.069	1	0.793	1.292	0.190	8.798
Variable(s) entered on step 3: AT12 popliteal.									
	GM 12 (absent)	−1.044	0.948	1.212	1	0.271	0.352	0.055	2.259
	AT 12 scarf (abnormal)	−.0287	1.043	0.076	1	0.783	0.751	0.097	5.796
	AT 12 popliteal (abnormal)	1.716	0.841	4.160	1	0.041	5.563	1.069	28.941
Variable(s) entered on step 4: AT12 axial.									
	GM12 (absent)	−1.504	1.060	2.014	1	0.156	0.222	0.028	1.774
	AT 12 scarf (abnormal)	0.085	1.101	0.006	1	0.938	1.089	0.126	9.415
	AT 12 popliteal (abnormal)	1.741	0.867	4.033	1	0.045	5.702	1.043	31.183
	AT 12 axial (abnormal)	0.901	0.878	1.053	1	0.305	2.462	0.440	13.770
Variable(s) entered on step 5: AT12 synthesis.									
	GM 12 (absent)	1.000	0.496	4.060	1	0.044	2.717	1.028	7.184
	AT 12 scarf (abnormal)	0.165	1.267	0.017	1	0.897	1.179	0.098	14.124
	AT 12 popliteal (abnormal)	−0.257	0.715	0.130	1	0.719	0.773	0.191	3.137
	AT 12 axial (abnormal)	0.432	0.501	0.742	1	0.389	1.540	0.577	4.112
	AT12 synthesis(abnormal)	1.160	0.263	19.499	1	0.001	3.191	1.907	5.341

Legend: AT 12—Amiel-Tison Exam at 12 weeks corrected age; CA—corrected age; GM 12—general movements—12 weeks corrected age.

**Table A11:** Binary logistic regression. Dependent variable: delayed walking. Independent variables: GM TEA, AT TEA scarf, AT TEA popliteal, AT TEA axial, AT TEA synthesis. Subgroup of preterm infants.

		B	S.E.	Wald	df	Sig.	Exp(B)	95.0% C.I. for EXP(B)	
								Lower	Upper
Variable(s) entered on step 1: GM TEA.									
	GM TEA (abnormal)	0.961	0.326	8.692	1	0.003	2.615	1.380	4.956
Variable(s) entered on step 2: AT TEA scarf.									
	GM TEA (abnormal)	1.128	0.347	10.584	1	0.001	3.091	1.566	6.100
	AT TEA scarf (abnormal)	−22.331	28,420.72	0.000	1	0.999	0.000	0.000	Not Computed
Variable(s) entered on step 3: AT TEA popliteal.									
	GM TEA (abnormal)	1.466	0.453	10.482	1	0.001	4.333	1.784	10.528
	AT TEA scarf (abnormal)	−21.673	28,420.72	0.000	1	0.999	0.000	0.000	.
	AT TEA popliteal (abnormal)	−0.996	0.728	1.873	1	0.171	0.369	0.089	1.538
Variable(s) entered on step 4: AT TEA axial.									
	GM TEA (abnormal)	1.478	0.470	9.894	1	0.002	4.382	1.745	11.004
	AT TEA scarf (abnormal)	−21.650	28,416.13	0.000	1	0.999	0.000	0.000	.
	AT TEA popliteal (abnormal)	−0.987	0.735	1.805	1	0.179	0.373	0.088	1.573
	AT TEA axial (abnormal)	−0.088	0.951	0.009	1	0.926	0.916	0.142	5.901
Variable(s) entered on step 5: AT TEA synthesis.									
	GM TEA (abnormal)	1.792	0.540	11.007	1	0.001	6.000	2.082	17.292
	AT TEA scarf (abnormal)	−41.027	31,152.97	0.000	1	0.999	0.000	0.000	.
	AT TEA popliteal (abnormal)	20.793	18,901.25	0.000	1	0.999	1 × 10^10^	0.000	.
	AT TEA axial (abnormal)	20.793	18,901.25	0.000	1	0.999	1 × 10^10^	0.000	.
	AT TEA synthesis (normal)	−22.584	18,901.25	0.000	1	0.999	0.000	0.000	.

Legend: AT 12—Amiel-Tison Exam at 12 weeks corrected age; CA—corrected age; GM 12—general movements—12 weeks corrected age. Not computed—the value could not be computed by the software—the model is not considered valid.

**Table A12:** Binary logistic regression. Dependent variable: delayed walking. Independent variables: GM 12, AT 12 scarf, AT 12 popliteal, AT 12 axial, AT 12 synthesis. Subgroup of preterm infants.

		B	S.E.	Wald	df	Sig.	Exp(B)	95.0% C.I. for EXP(B)	
								Lower	Upper
Variable(s) entered on step 1: GM12.									
	GM 12 (absent)	−1.204	0.658	3.345	1	0.067	0.300	0.083	1.090
Variable(s) entered on step 2: AT12 scarf.									
	GM 12 (absent)	−1.270	0.845	2.260	1	0.133	0.281	0.054	1.471
	AT 12 scarf (abnormal)	0.140	1.101	0.016	1	0.899	1.150	0.133	9.962
Variable(s) entered on step 3: AT12 popliteal.									
	GM 12 (absent)	−2.204	1.085	4.129	1	0.042	0.110	0.013	0.925
	AT 12 scarf (abnormal)	−0.167	1.121	0.022	1	0.882	0.846	0.094	7.621
	AT 12 popliteal (abnormal)	1.309	0.803	2.659	1	0.103	3.701	0.768	17.841
Variable(s) entered on step 4: AT12 axial									
	GM12 (absent)	−2.716	1.187	5.237	1	0.022	0.066	0.006	0.677
	AT 12 scarf (abnormal)	0.281	1.211	0.054	1	0.817	1.324	0.123	14.208
	AT 12 popliteal (abnormal)	1.274	0.811	2.469	1	0.116	3.574	0.730	17.498
	AT 12 axial (abnormal)	1.051	0.924	1.294	1	0.255	2.861	0.468	17.490
Variable(s) entered on step 5: AT12 synthesis.									
	GM 12 (absent)	1.000	0.496	4.060	1	0.044	2.717	1.028	7.184
	AT 12 scarf (abnormal)	0.165	1.267	0.017	1	0.897	1.179	0.098	14.124
	AT 12 popliteal (abnormal)	−0.257	0.715	0.130	1	0.719	0.773	0.191	3.137
	AT 12 axial (abnormal)	0.432	0.501	0.742	1	0.389	1.540	0.577	4.112
	AT12 synthesis(abnormal)	−0.296	0.792	0.139	1	0.709	0.744	0.157	3.515

Legend: AT 12—Amiel-Tison Exam at 12 weeks corrected age; CA—corrected age; GM 12—general movements—12 weeks corrected age.
