# Supplementary material for: General Movements Assessment and Amiel-Tison Neurologic Examination in Neonates and Infants: Correlations and Prognostic Values Regarding Neuromotor Outcomes
Source: Life (Basel). 2026 Jan 5;16(1):81. doi: 10.3390/life16010081 (PMC12843170; doi:10.3390/life16010081)
Supplement: Supplementary file 1 [file life-16-00081-s001.zip › life-4035157-supplementary.pdf]

Supplementary material S1.

Table S1. General characteristics of the non-premature (term) infants group.

Gestational age	Gender	Diagnosis	GM TEA	AT synthesis TEA	GM 12 w CA	AT synthesis 12 W CA	Sitting	Walking	CP
40	Male	Agenesis of the Corpus callosum	PR	Non-optimal	Fidgety	Optimal	Yes	On time	No
40	Male	Agenesis of the Corpus callosum	PR	Non-optimal	Fidgety	Non-optimal	Yes	On time	No
38	Male	Agenesis of the Corpus callosum	PR	Non-optimal	Fidgety	Non-optimal	Yes	On time	No
39	Male	Craniosynostosis – metopic	PR	Optimal	Fidgety	Optimal	Yes	On time	No
34	Female	Ventricular dilatation ex vaquo. Dysmorphic features – no diagnosis – parental refusal of testing	PR	Non-optimal	Fidgety	Non-optimal	Delayed	Absent at 2 years	Spastic bilateral GMFCS II
38	Female	Reuch Steidl Syndrome	Normal	Normal	Fidgety	Optimal	Delayed	Delayed	Spastic unilateral GMFCS II
36	Male	Ventricular dilatation. Hypoplasia of the corpus callosum. Cerebellar hypoplasia Mutation in the PPP2R1A gene on chromosome 19q13.4	CS	Non-optimal	Absent fidgety	Non-optimal	No	No	Spastic bilateral GMFCS V
37	Male	Subarachnoid cyst. Congenital cataracts. Ventricular septal defect.	PR	Non-optimal	Fidgety	Non-optimal	Delayed	Delayed	Spastic bilateral GMFCS II

Supplementary material S2 – Odds Ratios whole group

Table S2.1. Odds ratios for the risk of cerebral palsy (CP) – GM and Amiel Tison examinations. – whole group

	CP (n=15)	nonCP (n=55)	Chi Square test P	OR	CI95%
**GM TEA**			< 0.084	5.25	0.63-43.47
**Abnormal**	14 (93.3%)	40 (72.7%)			
**Normal**	1 (6.7%)	15 (27.3%)			
**AT TEA scarf**			< 0.008	5.58	3.34-9.32
**Abnormal**	3 (20.0%)	0 (0.0%)			
**Normal**	12 (80.0%)	55 (100.0%)			
**ATTEA popliteal**			< 0.010	5.14	1.51-17.49
**Abnormal**	6 (40.0%)	7 (12.7%)			
**Normal**	9 (60.0%)	16 (81.8%)			
**AT TEA axial**			< 0.026	4.57	1.24-16.81
**Abnormal**	10 (66.7%)	13 (23.6%)			
**Normal**	5 (33.3%)	48 (87.3%)			
**AT TEA synthesis**			< 0.003	6.46	1.87-22.35
**Non-optimal**	10 (66.7%)	13 (23.6%)			
**Optimal**	5 (33.3%)	42 (76.4%)			
**GM 12**			< 0.001	69.33	12.43-386.81
**Absent**	12 (80.0%)	3 (5.5%)			
**Fidgety**	3 (20.0%)	52 (94.5%)			
**AT12 scarf**			< 0.001	36.00	3.87-335.30
**Abnormal**	6 (40.0%)	1 (1.8%)			
**Normal**	9 (60.0%)	54 (98.2%)			
**AT12 popliteal**			< 0.001	8.00	2.27-28.22
**Abnormal**	10 (66.7%)	11 (20.0%)			
**Normal**	5 (33.3%)	44 (80.0%)			
**AT12 axial**			< 0.026	4.57	1.24-16.81
**Abnormal**	6 (40.0%)	7 (12.7%)			
**Normal**	9 (60.0%)	48 (87.3%)			
**AT12 synthesis**			< 0.001	9.75	2.42-39.25
**Non-optimal**	12 (80.0%)	16 (29.1%)			
**Optimal**	3 (20.0%)	39 (70.9%)			

Legend: AT TEA – Amiel Tison exam at Term Equivalent Age.; AT 12 – Amiel Tison exam at 12 weeks corrected age.; CI – confidence interval; GM TEA – general movements assessment at Term Equivalent Age. GM 12 general movements assessment at 12 weeks corrected Age. OR – Odds Ratio

Table S2.2. Odds Ratios for the risk of delayed sitting – whole group

	**Sitting delayed (n=15)**	**Sitting on time (n=55)**	**Chi Square test P**	**OR**	**CI 95%**
**GM TEA**			< 0.535	1.24	0.30-5.07
Abnormal	12 (80.0%)	42 (76.4%)			
Normal	3 (20.0%)	13 (23.6%)			
AT **TEA** scarf			< 0.114	8.31	0.70-98.77
Abnormal	2 (13.3%)	1 (1.8%)			
Normal	13 (86.7%)	54 (98.2%)			
**AT TEA popliteal**			< 0.043	3.50	1.04-11.74
**Abnormal**	7 (46.7%)	11 (20.0%)			
**Normal**	8 (53.3%)	44 (80.0%)			
AT TEA axial			< 0.103	2.094	0.79-10.88
Abnormal	5 (33.3%)	8 (14.5%)			
Normal	10 (66.7%)	47 (85.5%)			
AT TEA synthesis			< 0.058	3.05	0.94-9.87
Non-optimal	8 (53.3%)	15 (27.3%)			
Optimal	7 (46.7%)	40 (72.7%)			
**GM 12**			< 0.002	7.84	2.16-18.40
**Absent**	8 (53.3%)	7 (12.7%)			
**Fidgety**	7 (46.7%)	48 (87.3%)			
AT 12 scarf			< 0.163	3.19	0.63-16.17
Abnormal	3 (20.0%)	4 (7.3%)			
Normal	12 (80.0%)	51 (92.7%)			
AT 12 popliteal			< 0.104	2.56	0.79-8.36
Abnormal	7 (46.7%)	14 (25.5%)			
Normal	8 (53.3%)	41 (74.5%)			
**AT 12 axial**			< 0.026	4.57	1.24-16.81
**Abnormal**	6 (40.0%)	7 (12.7%)			
**Normal**	9 (60.0%)	48 (87.3%)			
**AT 12 synthesis**			< 0.019	4.11	1.22-13.82
**Non-optimal**	10 (66.7%)	18 (32.7%)			
**Optimal**	5 (33.3%)	37 (67.3%)			

Legend: AT TEA – Amiel Tison exam at Term Equivalent Age; AT 12 – Amiel Tison exam at 12 weeks corrected age.; CI – confidence interval; GM TEA – general movements assessment at Term Equivalent Age. GM 12 general movements assessment at 12 weeks corrected Age; OR – Odds Ratio.

Table S2.3. Risk of delayed/absent walking (Odds Ratio).- whole group.

	Walking delayed (n=18)	Walking on time (n=52)	Chi Square test P	OR	CI 95%
**GM TEA**			< 0.146	2.95	0.60-14.49
**Abnormal**	16 (88.9%)	38 (73.1%)			
**Normal**	2 (11.1%)	14 (26.9%)			
**AT TEA scarf**			< 0.015	4.47	2.86-6.98
**Abnormal**	3 (16.7%)	0 (0.0%)			
**Normal**	15 (83.3%)	52 (100.0%)			
**AT TEA popliteal**			< 0.009	4.78	1.48-15.40
**Abnormal**	9 (50.0%)	9 (17.3%)			
**Normal**	9 (50.0%)	43 (82.7%)			
**AT TEA axial**			< 0.069	3.21	0.91-11.36
**Abnormal**	6 (33.3%)	7 (13.4%)			
**Normal**	12 (66.7%)	45 (86.5%)			
**AT TEA synthesis**			< 0.004	5.24	1.67-16.48
**Non-optimal**	11 (61.1%)	12 (23.1%)			
**Optimal**	7 (38.9%)	40 (76.9%)			
**GM 12**			< 0.001	32.67	7.12-149.79
**Absent**	12 (66.7%)	3 (5.8%)			
**Fidgety**	6 (33.3%)	49 (94.2%)			
**AT 12 scarf**			< 0.010	9.62	1.67-55.31
**Abnormal**	5 (27.8%)	2 (3.8%)			
**Normal**	13 (72.2%)	50 (96.2%)			
**AT 12 popliteal**			< 0.002	6.60	2.04-21.31
**Abnormal**	11 (61.1%)	10 (19.2%)			
**Normal**	7 (38.9%)	42 (80.8%)			
**AT 12 axial**			< 0.016	4.88	1.37-17.43
**Abnormal**	7 (38.9%)	6 (11.5%)			
**Normal**	11 (61.1%)	46 (88.5%)			
**AT 12 synthesis**			< 0.001	9.50	2.67-39.79
**Non-optimal**	14 (77.8%)	14 (26.9%)			
**Optimal**	4 (22.2%)	38 (73.1%)			

Legend: AT TEA – Amiel Tison exam at Term Equivalent Age; AT 12 – Amiel Tison exam at 12 weeks corrected age.; CI – confidence interval. GM TEA – general movements assessment at Term Equivalent Age. GM 12 general movements assessment at 12 weeks corrected Age. OR – odds ratio.
